# Global secretome characterization of A549 human alveolar epithelial carcinoma cells during *Mycoplasma pneumoniae* infection

**DOI:** 10.1186/1471-2180-14-27

**Published:** 2014-02-07

**Authors:** Shuxian Li, Xuejing Li, Yingshuo Wang, Jun Yang, Zhimin Chen, Shigang Shan

**Affiliations:** 1 Department of Pediatric Pulmonology, The Children’s Hospital, Zhejiang University School of Medicine, Hangzhou, Zhejiang 310003, China; 2 Collaborative Innovation Center for Diagnosis and Treatment of Infectious Diseases, The First Affiliated Hospital, Zhejiang University, Hangzhou, Zhejiang 310013, China; 3 Department of Biomedicine, College of Biotechnology, Zhejiang Agriculture and Forestry University, Hangzhou, Zhejiang 311300, China

**Keywords:** *Mycoplasma pneumoniae*, Secretome, IL-33, Biological pathway

## Abstract

**Background:**

*Mycoplasma pneumoniae* (*M. pneumoniae*) is one of the major etiological agents for community-acquired pneumonia (CAP) in all age groups. The early host response to *M. pneumoniae* infection relies on the concerted release of proteins with various biological activities. However, no comprehensive analysis of the secretory proteins has been conducted to date regarding the host response upon *M. pneumoniae* infection.

**Results:**

We employed the liquid chromatography-tandem mass spectrometry (LC-MS/MS)-based label-free quantitative proteomic technology to identify and characterize the members of the human alveolar epithelial carcinoma A549 cell secretome during *M. pneumoniae* infection. A total of 256 proteins were identified, with 113 being differentially expressed (>1.5-fold change), among which 9 were only expressed in control cells, 10 only in *M. pneumoniae*-treated cells, while 55 were up-regulated and 39 down-regulated by *M. pneumoniae*. The changed expression of some of the identified proteins was validated by RT-PCR and immunoblot analysis. Cellular localization analysis of the secretome data revealed 59.38% of the proteins were considered as “putative secretory proteins”. Functional analysis revealed that the proteins affected upon *M. pneumoniae* infection were mainly related to metabolic process, stress response, and immune response. We further examined the level of one up-regulated protein, IL-33, in clinical samples. The result showed that IL-33 levels were significantly higher in the plasma and bronchoalveolar lavage fluid (BALF) of *M. pneumoniae* pneumonia (MPP) patients.

**Conclusions:**

The present study provided systematic information about the changes in the expression of secretory proteins during *M. pneumoniae* infection, which is useful for the discovery of specific biomarkers and targets for pharmacological intervention.

## Background

*Mycoplasma pnuemoniae* (*M. pneumoniae*) belongs to the class of the Mollicutes and is one of the smallest free-living organisms. It is a major cause of community-acquired pneumonia (CAP) worldwide in all age groups, and can also induce manifestations in extrapulmonary sites involving almost all organs of the human body [1,2]. With the exception of *M. pneumoniae* adherence to the host epithelium, molecular mechanisms underlying the pathogenesis of *M. pneumoniae* infection has long been a mystery [1]. Subsequent to cytadherence, *M. pneumoniae* is believed to cause disease in part through the generation of peroxide [3] and the induction of inflammatory reaction including cytokine productions (e.g. IL-8, TNF-α, and IL-1β) [4]. Simultaneously, autoimmunity developed after *M. pneumoniae* infection likely contributes to the extrapulmonary complications. For example, anti-GM1 and galactocerebroside antibodies are the primary autoantibodies implicated in the ascending paralysis of Guillain-Barre syndrome and in encephalitis associated with *M. pneumoniae*[5,6]. Although toxin had not been considered as part of the *M. pneumoniae* repertoire in previous studies, recent evidence suggested otherwise. A newly identified exotoxin of *M. pneumoniae*, named community-acquired respiratory distress syndrome toxin (CARDS TX), which has ADP-ribosylating and vacuolating activity, has been suggested to be responsible for eliciting extensive vacuolization and ciliostasis of host cells [7]. Thus, the pathophysiology of *M. pneumoniae* infection is likely to be complex and multifactorial, and the underlying molecular mechanisms should involve a large number of genes/proteins participating in various biological pathways [3,8,9]. High-throughput technologies including genomics and proteomics can comprehensively and quantitatively decipher gene/protein expression, and therefore, are useful tools in the study of complex systems under the influence of biological perturbations, such as pathogen-host interaction [10]. Previously, using a proteomic approach, we had analyzed *M. pneumoniae*-induced protein expression profile using whole cell lysates, and identified the redox regulatory pathway as a key target during *M. pneumoniae* infection [3]. However, as noted above, *M. pneumoniae*-induced immune response is important for *M. pneumoniae* pathogenesis, and many factors involved in the immune response, such as the cytokines, are so-called secretory proteins, which are part of the “secretome” [11].

Secretome proteins include extracellular matrix proteins, growth factors, cytokines and hormones, and other soluble mediators. It is known that secretory proteins are important for many physiological processes [11,12]. For example, the matrix metalloproteinases (MMPs), as extracellular matrix-degrading enzymes, are essential regulators of the cell’s microenvironment governing cell fate and function, such as cell migration, proliferation, apoptosis, invasion and development [13]. Moreover, changes in secretory proteins can reflect different conditions of the cells or tissues. For instance, Lietzen *et al.* revealed dramatic changes in secretome of macrophages, such as robust secretion of different danger-associated molecular patterns (DAMP), in response to influenza A infection [10]. Arturo *et al.* found that muscle secretion pattern varied according to fiber-type constituents (e.g. slow-oxidative compared to fast-glycolytic muscle), and the secretome could be affected by endurance exercise training [14]. Consequently, secretome represent an important source for biomarker and therapeutic target discovery [12]. For that importance, secretomics, a branch of proteomics, focusing on analyzing the profile of all proteins secreted from cells or tissues, has been developed in recent years [15].

In addition, recent studies have showed that secretory proteins are also important for certain disease conditions. For example, dysregulation of adipocytokines (e.g. TNF-α, plasminogen activator inhibitor type 1 (SERPINE1), heparin-binding epidermal growth factor-like growth factor) and adiponectin contributes to the development of a variety of cardiovascular disease [16]. Similarly, secretory proteins also play a role in infectious disease. For instance, changes in the expression of secretory proteins during latent human cytomegalovirus (HCMV) infection have profound effects on the regulation of the host immune response, such as recruitment of CD4+ T cells by increasing the expression of CC chemokine ligand 8 (CCL-8) [17]. Also, the secreted IFN-induced proteins (e.g. interferon-induced tetratricopeptide proteins 2 (IFIT2), IFIT3, signal transducer and activator of transcription 1 (STAT1)) were indicated to have important extracellular antiviral functions during Herpes simplex virus 1 (HSV-1) infection [18]. Together, these data indicate the important role of secretory proteins in host-pathogen interaction. However, although *M. pneumoniae* infection is a common cause of respiratory disease, secretome change during *M. pneumoniae* infection had not been thoroughly investigated.

Airway epithelial cells form the first line of defense against exposure to infectious agents. Epithelial cells are known to kill or neutralize microorganisms through the production of enzymes, permeabilizing peptides, collectins, and protease inhibitors during the innate immune response [19]. Epithelial cells are also essential in regulating adaptive immune responses in the airways by expressing pattern-recognition receptors (PRRs) to trigger host defense response, by activating dendritic cells to regulate Ag sensitization, and by releasing cytokines to recruit effector cells [4,19,20]. Thus, airway epithelial cells are important for the initiation, maintenance, and regulation of both innate and adaptive immune responses, as well as modulating the transition from innate to adaptive immunity. As the interaction of *M. pneumoniae* with respiratory epithelial cells is a critical early step of pathogenesis [21], and considering the importance of secretory proteins, a large-scale study on *M. pneumoniae*-induced protein secretion will help elucidate the molecular mechanisms related to *M. pneumoniae* infection.

Therefore, in the current study, we applied liquid chromatography-tandem mass spectrometry (LC-MS/MS) based label-free quantitative shotgun proteomics approach for global profiling of the *M. pneumoniae*-infected human alveolar epithelial carcinoma A549 cell secretome, in an effort to provide a better view of host-pathogen interaction and identify novel molecules/biomarkers for *M. pneumoniae* infection. As reported here, we have identified 113 proteins affected by *M. pneumoniae* infection. Furthermore, we evaluated the clinical application of one identified protein, IL-33, as a “proof of concept” example, and the result showed that it could help to distinguish *M. pneumoniae* pneumonia (MPP) patients from non-*M. pneumoniae* patients.

## Results

### Label-free quantitative shotgun proteomic analysis of cell secretome upon *M. pneumoniae* infection

**Figure 1:**
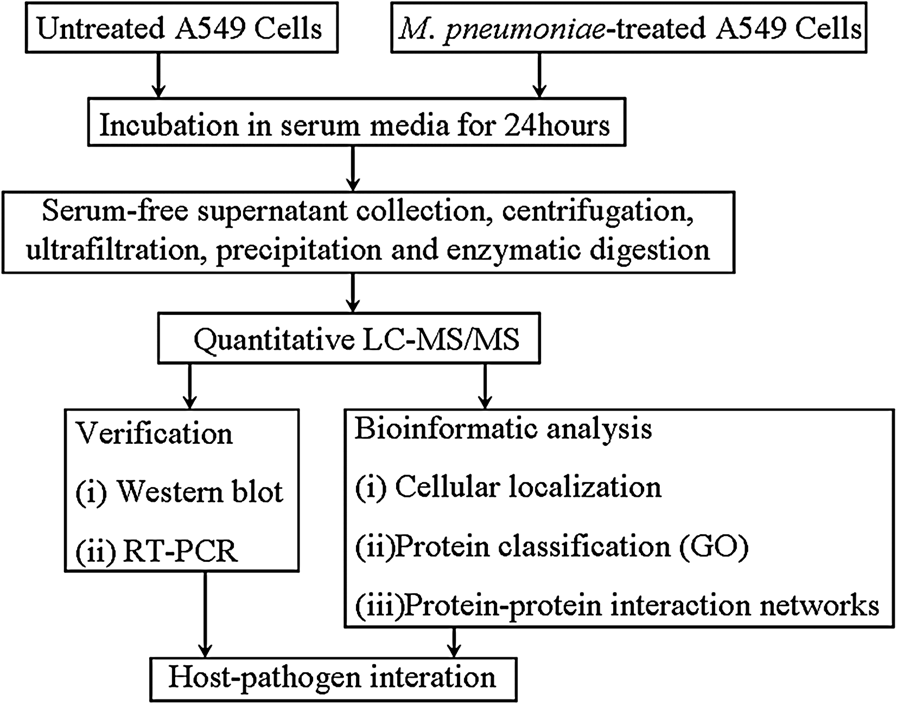
Workflow chart of the experimental design.

The study design is outlined in Figure 1. Both cell viability and apoptosis assay revealed that serum free medium (SFM) did not significantly affect cell integrity and secretion capacity within 24 h (see Additional files 1 and 2: Figures S1 and S2), and thus serum-free culture for 24 h was chosen as the time point for secretome collection.

Based on the LC-MS/MS data, 233 proteins were identified in control A549 cells, with 187 being identified from all three biological replicates (see Additional file 3: Figure S3A), indicating a relatively good reproducibility. Similarly, 237 proteins were identified in *M. pneumoniae*-infected A549 cells, with 199 being identified from all three biological replicates (see Additional file 3: Figure S3B). Thus, a total of 256 proteins were identified, among which 214 proteins were detected in both groups, with 19 and 23 proteins being uniquely secreted by control cells and *M. pneumoniae*-infected cells, respectively (see Additional file 3: Figure S3C). Complete protein identification lists for control and *M. pneumoniae*-infected cells were provided in Additional files 4 and 5: Datasheet S1 and Table S1.

For the identified proteins, label-free quantitative comparison performed by DeCyder™ MS Differential software revealed that 113 proteins were significantly affected by *M. pneumoniae* infection (fold difference ≥1.5 or ≤0.67) (see Additional file 6: Table S2). Specifically, there were 65 up-regulated and 48 down-regulated proteins in *M. pneumoniae*-infected A549 cells, among which 10 were uniquely expressed in *M. pneumoniae*-treated A549 and 9 in control A549 cells. For all 113 differential proteins, the number of peptides for each protein used for quantification varied from 1 to 13. Among them, 33 proteins were quantified on the basis of two or more peptides, with average coefficient of variation (CV) of the fold changes for peptides as 16.80% (range from 0.00% to 39.21%, see Additional file 6: Table S2), demonstrating a rational reproducibility of the quantitative data. The rest 80 proteins were quantified with only one peptide by the DeCyder software.

### Validation of proteins with changed expression during *M. pneumoniae* infection

**Figure 2:**
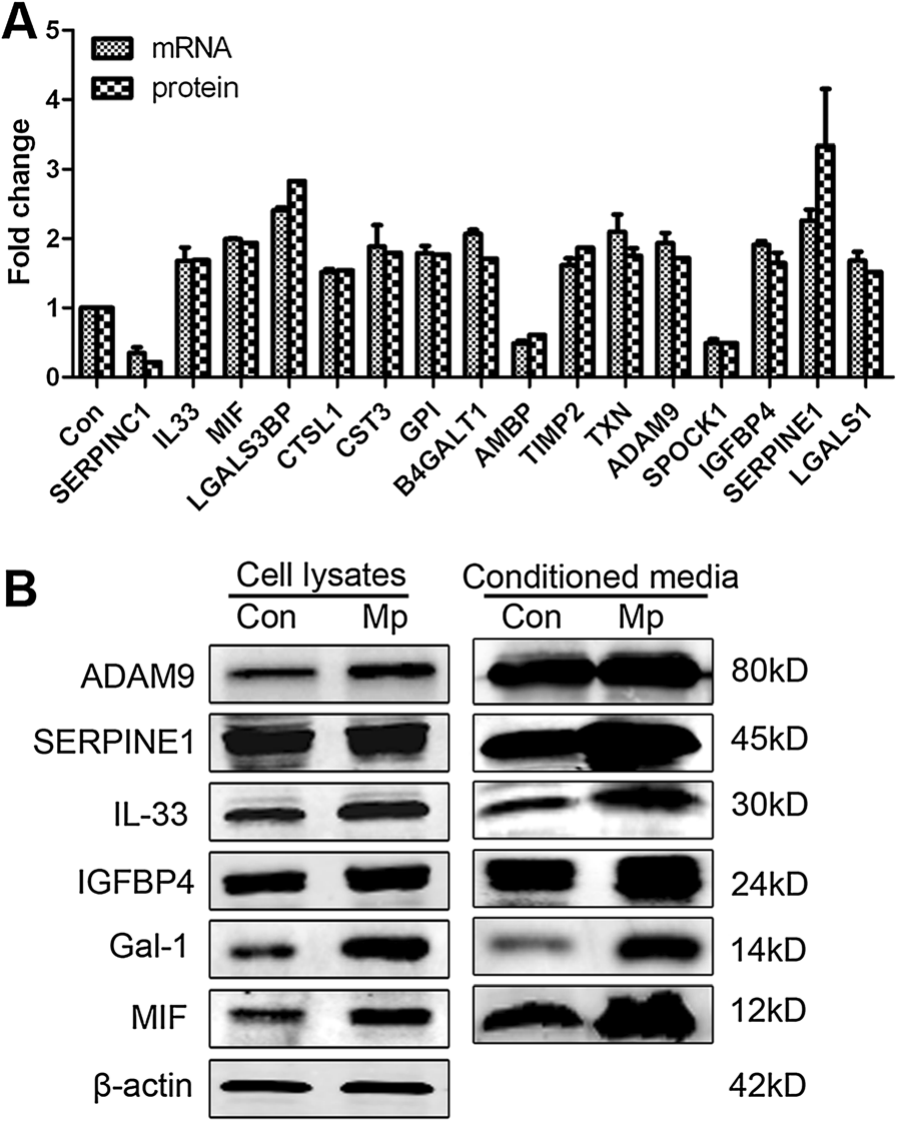
**Verification of up- or down-regulated proteins during *M. pneumoniae *infection. (A)** RT-PCR analysis and quantitative analysis data of 16 secertory proteins during *M. pneumoniae* infection. compared to control (*p <* 0.05); data are presented as means ± SD. **(B)** Western blot analysis for 6 secretory proteins from total cell lysates and culture supernatants. Representative images were from three independent experiments performed in duplicate. β-actin was used as internal control for total cell lysates.

To verify the proteomic results, real-time PCR and Western blot analysis were performed on several identified proteins. The real-time PCR results demonstrated that the gene expression levels of 16 secretory proteins exhibited the same trend of changes as the quantitative MS results (Figure 2A). Also, Western blot data showed that protein levels of six secretory proteins were significantly increased in the CM and total cell lysates after *M. pneumoniae*-infection, which were consistent with the proteomic results (Figure 2B). Therefore, from the RT-PCR and Western blot results, we found that these six secretory proteins (ADAM9, SERPINE1, IL-33, IGFBP4, Gal-1, MIF) were overexpressed in *M. pneumoniae*-infected A549 cells at mRNA and protein levels.

### Cellular localization of the identified proteins

The 256 identified proteins were first categorized as classical secretory proteins or non-classical secretory proteins based on SingalP and SecretomeP analysis. Of the 256 proteins, 83 were categorized as classical secretory proteins and 69 as non-classical secretory proteins (see Additional file 5: Table S1). To determine whether some of the proteins could also be released *via* exosomes, the Exocarta exosome database were searched [22]. The results showed that among the proteins identified, 190 proteins were also listed in the exosomal protein database (see Additional file 5: Table S1).

**Figure 3:**
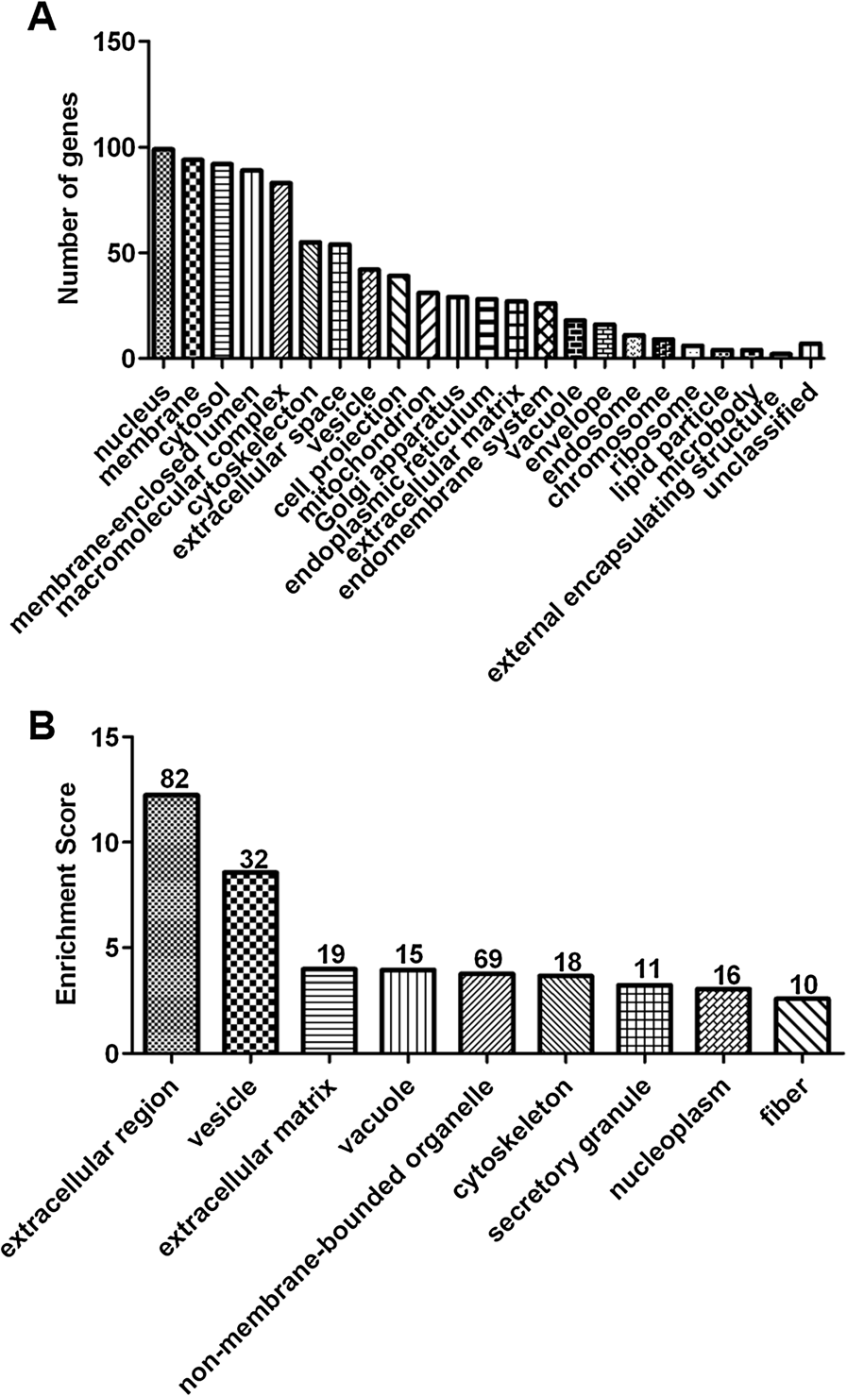
**Cellular localization of identified proteins. (A)** Distribution of the identified proteins based on gene ontology (GO) annotations. **(B)** Enrichment score of GO cellular component categories. DAVID 6.7 was used to analyze the GO classification of the identified proteins. Function annotation clustering was used to classify similar annotation terms together, and the enrichment score for each group was used to rank the overall over-representation of annotation terms. The higher the enrichment score, the more important were the members of the annotation cluster.

**Figure 4:**
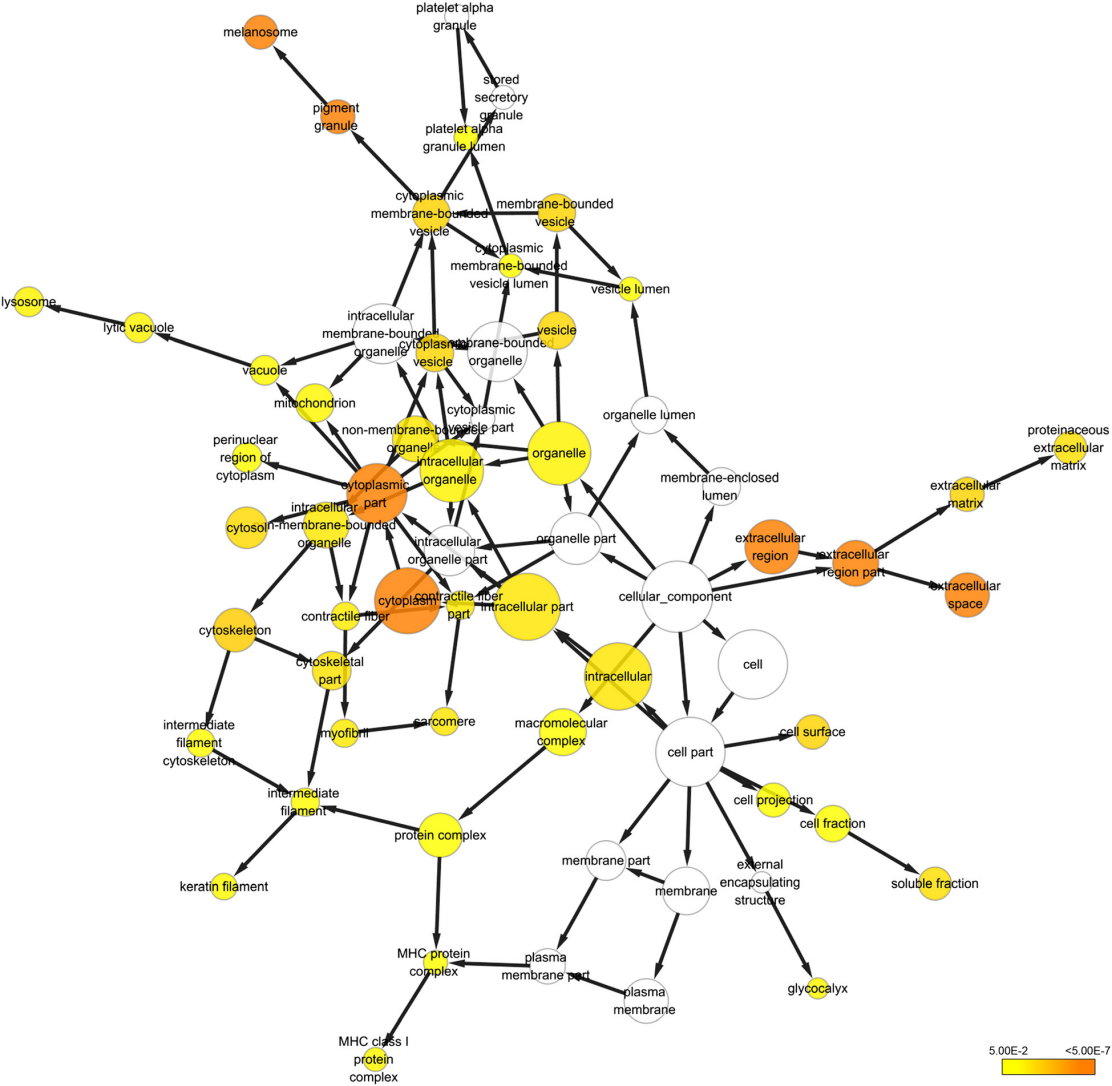
**Functional gene ontology (GO) analysis of cellular compartment distribution of the clusters of proteins that were up-regulated by *M. pneumoniae *treatment.** Over-representation of GO categories was analyzed using the Biological Networks Gene Ontology plugin (BINGO, version 2.44). Over-representation statistics were calculated by using the hypergeometric analysis and Benjamini & Hochberg False Discovery Rate (FDR) correction. Only categories that are significantly enriched after correction are represented. The color scales indicate the *p* value range for over-representation. The node size is proportional to the number of proteins annotated with the GO term.

We next analyzed the ontology of the identified proteins based on cellular compartment. The results showed majority of the proteins belong to more than one GO class (Figure 3). Most of the proteins have a nuclear distribution (Figure 3A). Functional annotation clustering analysis by DAVID 6.7 showed that when considering only cellular compartment distribution, the proteins of the extracellular region, vesicle and extracellular matrix were over-represented (enrichment score (ES) of 12.24, 8.57, and 3.98, respectively) (Figure 3B). Similarly, the classification based on the cellular organelle of the differentially expressed proteins also showed that *M. pneumoniae* infection did not induce protein secretion from any specific cell organelle, but rather, altered the overall secretion of proteins from all the main organelles, including mitochondrion and lysosome (Figure 4 and see Additional file 7: Figure S4A). Enrichment in proteins residing in the extracellular region, especially extracellular matrix, extracellular space, and membrane-bound vesicle was observed (Figure 4 and see Additional file 7: Figure S4A). Moreover, when *p* value < 0.05 was set as the threshold, 46 GO terms for up-regulated proteins and 10 for down-regulated proteins were obtained, respectively (Figure 4 and see Additional file 7: Figure S4A).

### Functional classification of the differentially expressed secretory proteins

To better understand the nature of the differentially expressed proteins, the KEGG database was used for pathway analysis, which evaluates the relative importance of the change in a pathway/function in response to treatment and/or change in physiological state. Eleven pathways were listed in the KEGG database (*p <* 0.1) after *M. pneumoniae* infection, of which 8 were significantly over-represented (*p <* 0.05) (Table 1). The significantly over-represented KEGG pathways were related to metabolism, infection, and proliferation (Table 1).

**Table 1:** **KEGG analysis of differential expressed protein after *Mycoplasma pneumoniae *** **infection**

**Category**	**Term**	**Count**	**%**	***p*** **value**	**Genes**
KEGG_PATHWAY	hsa00620:Pyruvate metabolism	6	5.31	1.46E-04	3939, 4191, 4190, 231, 5315, 3945
KEGG_PATHWAY	hsa00010:Glycolysis/Gluconeogenesis	6	5.31	9.95E-04	3939, 7167, 2023, 5315, 3945, 2821
KEGG_PATHWAY	hsa04114:Oocyte meiosis	7	6.19	2.83E-03	10971, 7529, 5501, 801, 7534, 7532, 7531
KEGG_PATHWAY	hsa00030:Pentose phosphate pathway	4	3.54	3.92E-03	2539, 7086, 2821, 5226
KEGG_PATHWAY	hsa00270:Cysteine and methionine metabolism	4	3.54	9.38E-03	3939, 191, 3945, 2805
KEGG_PATHWAY	hsa04722:Neurotrophin signaling pathway	6	5.31	2.17E-02	10971, 7529, 801, 7534, 7532, 7531
KEGG_PATHWAY	hsa00480:Glutathione metabolism	4	3.54	2.65E-02	2950, 2539, 2936, 5226
KEGG_PATHWAY	hsa05130:Pathogenic Escherichia coli infection	4	3.54	3.72E-02	10971, 7534, 3875, 10376
KEGG_PATHWAY	hsa04810:Regulation of actin cytoskeleton	7	6.19	5.89E-02	80310, 4478, 5501, 2335, 81, 5216, 1072
KEGG_PATHWAY	hsa00051:Fructose and mannose metabolism	3	2.65	7.14E-02	7167, 231, 57016
KEGG_PATHWAY	hsa04110:Cell cycle	5	4.42	7.82E-02	10971, 7529, 7534, 7532, 7531

In addition, an enrichment analysis was performed by BiNGO for the differentially expressed proteins. For the up-regulated proteins, GO analysis revealed 19 molecular function GO categories associated with oxidoreductase activity, 18 related to protein binding, and 16 linked to enzyme regulator activity (see Additional file 7: Figure S4B), while for the down-regulated proteins, 54 GO terms were identified, which were mainly associated with enzyme inhibitor activity and hydrolase activity (see Additional file 7: Figure S4C).

Biological processes analysis of up-regulated proteins led to the identification of functional groups related to monosaccharide catabolic process, inflammatory response, cell redox homeostasis, and defense response (see Additional file 7: Figure S4D). For the down-regulated proteins, system development seemed to be the most relevant biological process (see Additional file 7: Figure S4E). Based on the BiNGO biological process analysis, we identified 17 up-regulated proteins that were involved in immune response, and 36 up-regulated proteins might act as endogenous danger signals [23,24] (Table 2).

**Table 2:** **Immune response and stress related proteins that were up-regulated by *Mycoplasma pneumoniae *** **infection**

**GO-ID**	**Description**	**Genes in test set**
**Stress related proteins and functions**		
9611	Response to wounding	B4GALT1|YWHAZ|PROCR|LGALS1|CLU|SERPINE1|NP|IGFBP4|FN1|MIF
6950	Response to stress	B4GALT1|YWHAZ|LGALS1|CLU|CST3|PRDX1|NP|TPM4|MIF|PFN1|LGALS3BP|PROCR|AKR1B1|SERPINE1|HSPB1|CTSD|IGFBP4|FN1|ADAM9
50896	Response to stimulus	LDHA|YWHAZ|CLU|PRDX1|NP|TPM4|B2M|MIF|PFN1|APP|LGALS3BP|GOT1|PROCR|TGFBI|SERPINE1|ADAM9|ENO1|FN1|B4GALT1|LGALS1|CST3|GPI|NPC2|AKR1B1|CFL1|CTSD|HSPB1|IGFBP4|CALM1
9615	Response to virus	NPC2|CLU|CFL1|HSPB1|ENO1
55114	Oxidation reduction	ALDH1A1|LDHB|LDHA|AKR1B10|AKR1B1|TXN|PGD|PRDX4|TXNRD1|PRDX1
51707	Response to other organism	NPC2|CLU|CFL1|SERPINE1|HSPB1|ENO1|B2M
6979	Response to oxidative stress	CLU|SERPINE1|PRDX1|TPM4|ADAM9
42221	Response to chemical stimulus	YWHAZ|LGALS1|CLU|PRDX1|NP|TPM4|B2M|MIF|PFN1|GOT1|SERPINE1|CFL1|HSPB1|CALM1|ADAM9
9607	Response to biotic stimulus	NPC2|CLU|CFL1|SERPINE1|HSPB1|ENO1|B2M
10035	Response to inorganic substance	SERPINE1|PRDX1|CALM1|ADAM9|B2M
10033	Response to organic substance	PFN1|GOT1|LGALS1|CLU|CFL1|SERPINE1|HSPB1|MIF|ADAM9|B2M
42493	Response to drug	YWHAZ|LGALS1|NP|MIF|B2M
302	Response to reactive oxygen species	SERPINE1|PRDX1|ADAM9
**Immune response related proteins and functions**		
6952	Defense response	B4GALT1|PFN1|LGALS3BP|YWHAZ|CLU|SERPINE1|CST3|PRDX1|IGFBP4|FN1|MIF
6954	Inflammatory response	B4GALT1|YWHAZ|CLU|IGFBP4|FN1|MIF
50900	Leukocyte migration	B4GALT1|MSN|MIF
2376	Immune system process	B4GALT1|GPI|YWHAZ|PROCR|CLU|MSN|NP|PRDX1|MIF|ADAM9|B2M
2441	Histamine secretion involved in inflammatory response	YWHAZ
2477	Antigen processing and presentation of exogenous peptide antigen via MHC class Ib	B2M
2481	Antigen processing and presentation of exogenous protein antigen via MHC class Ib, TAP-dependent	B2M
2349	Histamine production involved in inflammatory response	YWHAZ
35491	Positive regulation of leukotriene production involved in inflammatory response	SERPINE1
35490	Regulation of leukotriene production involved in inflammatory response	SERPINE1
34241	Positive regulation of macrophage fusion	ADAM9
6955	Immune response	GPI|YWHAZ|PROCR|CLU|NP|PRDX1|MIF|B2M
2443	Leukocyte mediated immunity	YWHAZ|CLU|PRDX1
2684	Positive regulation of immune system process	CLU|SERPINE1|NP|MIF|B2M

### Protein-protein interaction network analysis during *M. pneumoniae* infection

**Figure 5:**
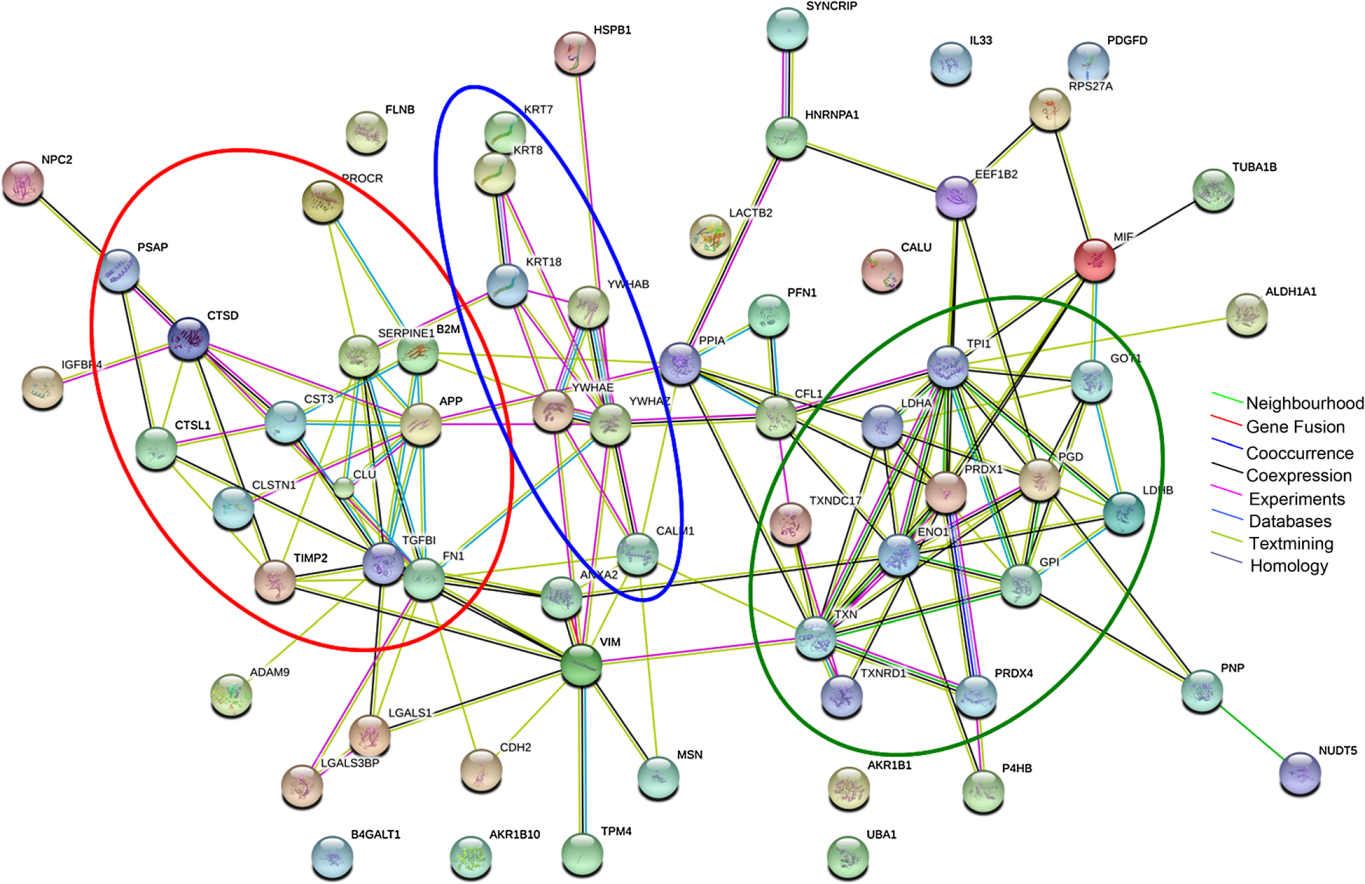
**Protein interaction network analysis of the up-regulated proteins in *M. pneumoniae*-treated A549 cells.** Using protein interaction network analysis tool (STRING database), three networks of the associated proteins were found among the up-regulated proteins. These included the network for stress response proteins (red circle), signaling pathway associated proteins (blue circle), and cellular metabolic proteins (green circle). Different line colors represent the types of evidence for the association.

**Figure 6:**
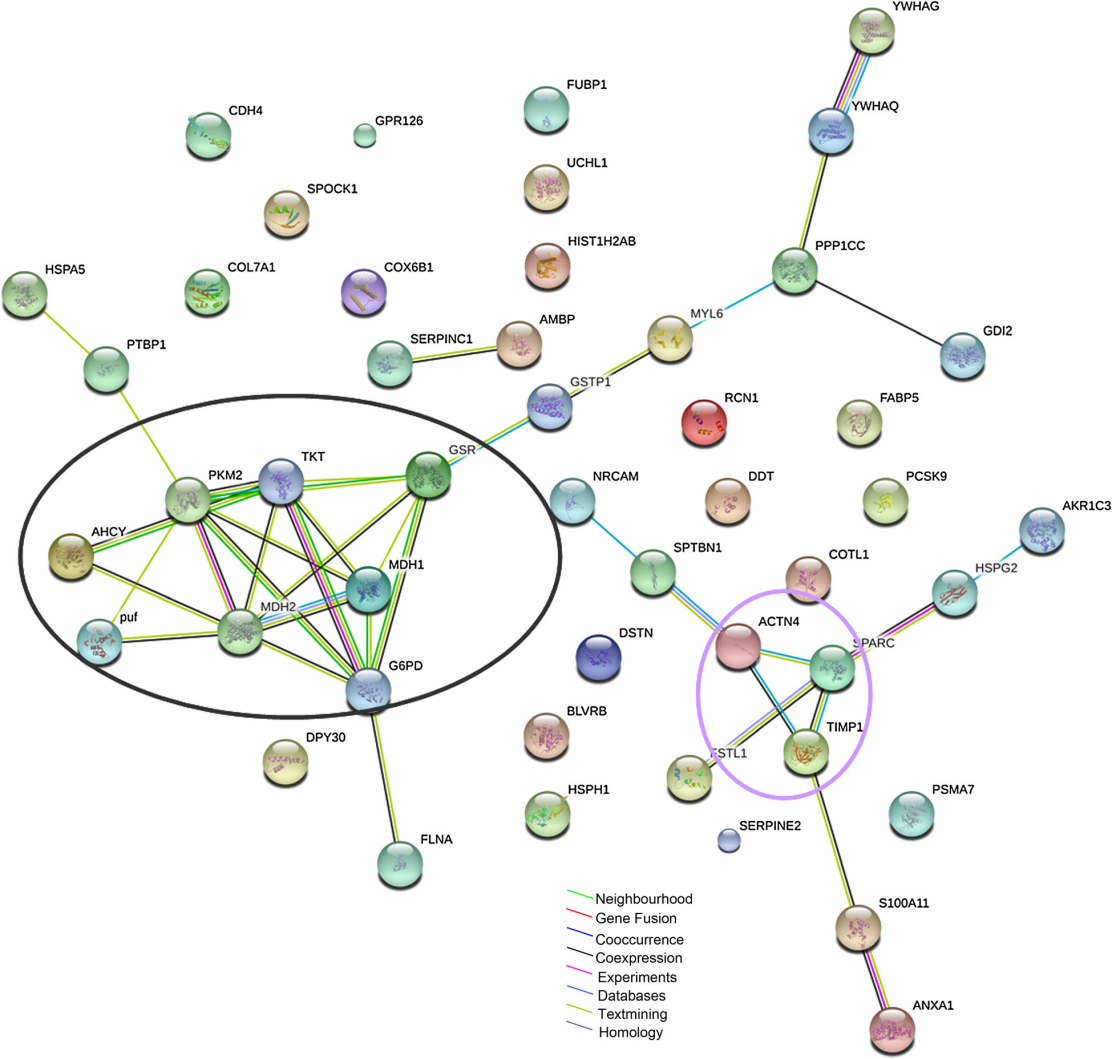
**Protein interaction network generated with STRING software for down-regulated proteins in *M. pneumoniae*-treated A549 cells.** Two major networks, e.g., glucose catabolic proteins (black circle) and biological process negative regulation associated proteins (purple circle) were found. Different line colors represent the types of evidence for the association.

As mentioned earlier, pathogen-host interaction is a very complex process and many proteins are involved. Also, biological association network changes in protein expression are not isolated events [25]. Therefore, in this study, we want to know how differentially expressed proteins interact with each other and how they affect cell’s function during *M. pneumoniae* infection. The biological associations among the differentially expressed proteins were investigated using the STRING software. The predicted protein-protein associations were queried through a vast number of databases derived in different ways (e.g. experimentally determined interactions, protein neighborhood data, or data acquired via text mining) [26]. As shown in Figure 5, for the 65 up-regulated proteins, three main networks of protein interactions were identified, including stress response proteins (red circle), signaling pathway associated proteins (blue circle), and cellular metabolic proteins (green circle). For the 48 down-regulated proteins, two major networks of the associated proteins were found, including the glucose catabolic proteins (black circle) and biological process negative regulation associated proteins (purple circle) (Figure 6).

### Clinical examination of IL-33 expression in plasma and BALF

**Figure 7:**
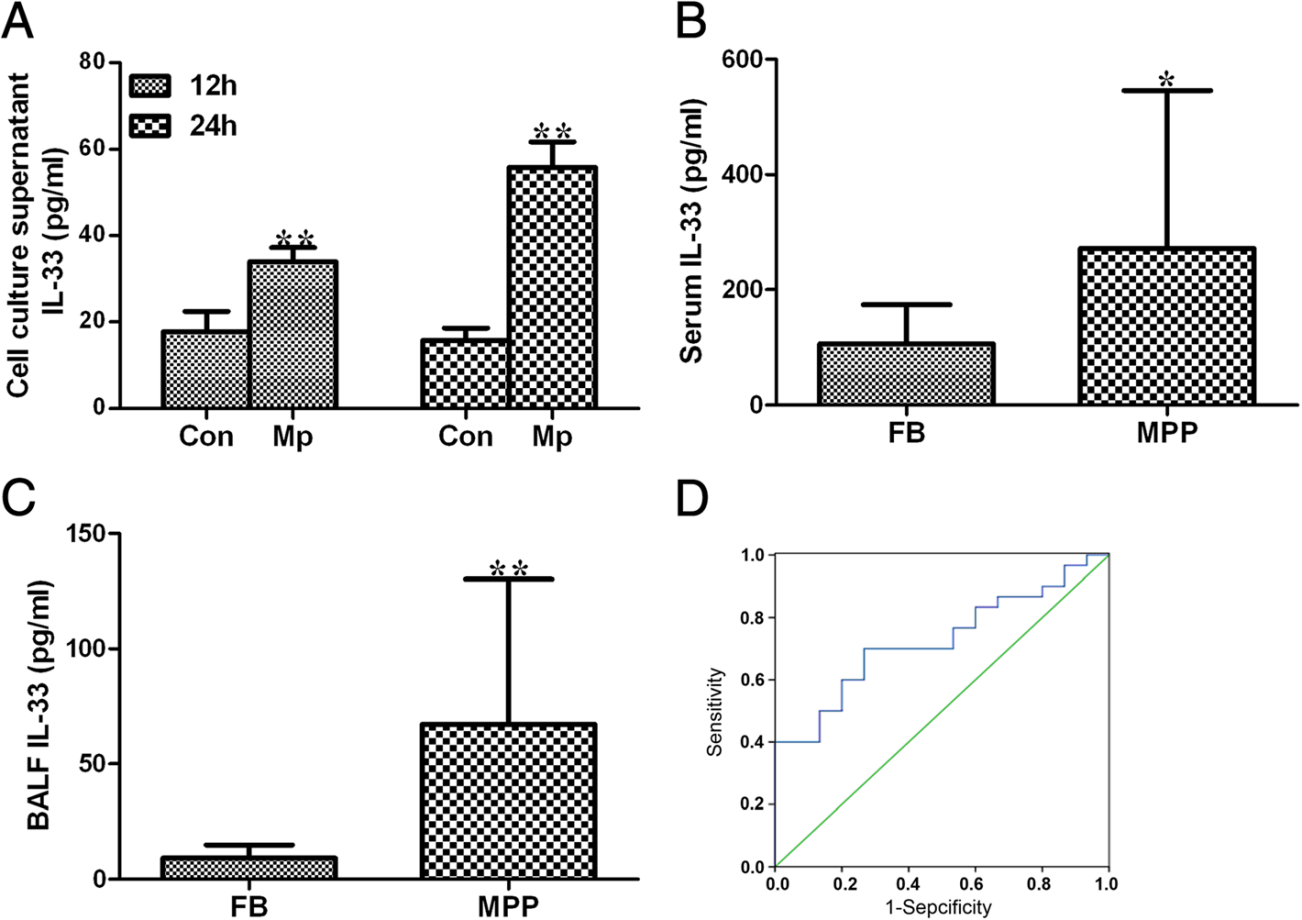
***M. pneumoniae *infection induces IL-33 expression. (A)** A549 cells were treated with *M. pneumoniae* for 12 and 24 h, and IL-33 levels in the supernatants were measured by ELISA. Data are presented as means ± SD from at least three independent experiments. **, *p <* 0.01, compared with untreated A549 cells. **(B)** Concentration of IL-33 in patient plasma samples. **(C)** Concentration of IL-33 in bronchoalveolar lavage fluid (BALF) samples. Samples were obtained from patients with foreign body (FB, control, n = 15) and patients with *M. pneumoniae* pneumonia (MPP, n = 30). Data are presented as mean ± SD, significance was determined by Mann–Whitney U test. *, *p <* 0.05; **, *p <* 0.01, compared with FB. **(D)** ROC curve analysis of the diagnostic efficacy of IL-33 between MPP patients and control (AUC = 0.727).

To explore the potential clinical application of secretomic data, we selected one of the identified proteins, IL-33, and examined its expression in the plasma and BALF of MPP patients, using age-matched patients with foreign body as comparison cohort. Patient information was listed in Table 3. First it was shown that IL-33 secretion was induced in A549 cells by *M. pneumoniae* infection (Figure 7A). Results from the measurements of patient samples also showed that IL-33 level was significantly higher in both plasma and BALF of MPP patients than those in patient with foreign body (Figure 7B and 7C). To further evaluate whether the increased plasma IL-33 levels had any potential clinical significance as a possible biomarker for helping distinguish MPP patients from controls, a receiver operating characteristic (ROC) curve was constructed by plotting sensitivity vs. specificity. The area under the ROC curve (AUC), a commonly used indicator for estimating the diagnostic efficacy of a potential biomarker, was subsequently calculated. For differentiating MPP patients from controls, the AUC was determined to be 0.727 (95% confidence interval, 0.580-0.873) for plasma IL-33 (Figure 7D). When a cutoff value of 129.08 pg/ml was set for plasma IL-33, the sensitivity and specificity for discriminating MPP patients from controls were 70.0% and 73.3%, respectively.

**Table 3:** Clinical information of patients with MPP or FB

**Characteristics**	**FB (n = 15)**	**MPP (n = 30)**	***p*** **value**
Age (years)	4.88 ± 3.58	5.78 ± 2.46	0.326
Gender (male/female)	9/6	16/14	0.671
Peripheral leukocyte (×10^9^ cells/L)	7.00 ± 1.64	9.06 ± 4.10	0.102
Peripheral neutrophil (%)	46.95 ± 20.89	63.90 ± 16.20	0.004
BAL macrophage (%)	84.73 ± 6.45	66.53 ± 13.71	< 0.001
BAL lymphocyte (%)	9.73 ± 3.88	11.93 ± 6.39	0.229
BAL neutrophil (%)	5.53 ± 3.68	20.73 ± 13.47	< 0.001
BAL eosinophil (%)	0.20 ± 0.41	0.83 ± 2.35	0.309

Data were expressed as mean ± SD. These variables were compared using Student’s t-test or Mann–Whitney U test.

## Discussion

By using comprehensive MS-based proteomics combined with label-free quantitation algorithms, we examined the secretome of *M. pneumoniae*-infected and uninfected A549 cells. This proteomic approach allows the simultaneous observation of alternation in protein expression, which may represent a forecast to disease development or a consequence of the disease [27]. As reported here, a total of 256 proteins were identified, among which 113 were differentially secreted by *M. pneumoniae*-infected A549 cells versus control. This result is similar to a study conducted by Brioschi et al., in which 273 proteins were identified and 112 differentially expressed in the endothelial cell secretome upon reductase inhibitor treatment [28].

Among the identified proteins, 152 proteins were designated as putative secretory proteins by using SignalP and SecretomeP. Interestingly, 69 out of the 152 proteins were categorized as non-classical secretory proteins, suggesting that the unconventional protein release is also a major mechanism. More importantly, as exosomal release is also regarded as a non-classical secretion mechanism [29], it was shown that 74% (190 out of 256) of the identified proteins in our study can be found in the ExoCarta database, highlighting a critical role for exosome in cell-cell communication [22]. In summary, up to 92% (236 out of 256) of the identified proteins could be transported to the extracellular space by at least one of the above mechanisms. Since no significant apoptosis or necrosis was observed in our study (see Additional file 2: Figure S2), those proteins, which were not classified as secretory proteins using the computational approach (SignalP and SecretomeP), should be released mainly by intracellular secretion (e.g. exosome) rather than cell lysis [30]. Furthermore, among the 113 differentially expressed proteins, about 80% (91) were found in the ExoCarta database, suggesting that exosomal protein release might be a major mechanism by which *M. pneumoniae*-infected cells communicate with other cells. Similarly, exosome-mediated release of proteins in influenza A virus-infected human macrophages has also been reported, underlining the importance of the exosome-mediated non-classical pathway in cell-to-cell communication during microbial infection [10].

Based on STRING bioinformatics analysis, several clusters of proteins were identified (Figure 5 and 6), suggesting that these proteins often act in cooperation with each other rather than alone during *M. pneumoniae* infection. Furthermore, the functions of those differential expressed proteins were found to be mainly associated with biological processes including immune response, metabolic process, and stress response (see Additional file 7: Figure S4D and S4E). Indeed, a number of studies have highlighted the importance of host-dependent inflammatory response to *M. pneumoniae* infection, such as IL-12 and IFN-γ production, as well as the Th1 type T-cell responses in a mouse model [4,31-34]. Previously we have also shown that the reactive oxygen species (ROS) induced by *M. pneumoniae* infection attributed in part to the cytopathology of the respiratory epithelium [3], and *M. pneumoniae* infection could influence host’s sphingolipid metabolism, including the generation of new ceramide and sphingomyelin species [35]. These reports, together with many other reports, supported the finding from this secretomic study that *M. pneumoniae* infection systematically alters the biological process of the host, which may partially explain the wide clinical manifestation of *M. pneumoniae* infection [2].

Cells under stress are known to actively secrete or passively release endogenous danger signal molecules, which include proteins and other endogenous molecules, such as ATP and uric acid [23,36]. Interestingly, we have found 36 out of the 113 differentially expressed proteins were associated with stress and may act as endogenous danger signals (Table 2) [23,24], including heat shock protein beta-1 (HSPB1), galectin-1 (Gal-1), galectin-3-binding protein (LGALS3BP), SERPINE1, disintegrin and metalloproteinase domain-containing protein 9 (ADAM9), peroxiredoxin-4 (PRDX4), and PRDX1. Several of these danger signal proteins, such as HSPs, galectins, and redox-related members, were also secreted during influenza A virus or HSV-1 infection of human macrophages [10,18]. Therefore, the secretion of such danger signal proteins might be a general host response to pathogen infection. Some of these danger signal molecules were involved in regulating the cellular oxidative status, such as ADAM9, Gal-1 and SERPINE1 [37-39]. In line with such observation, *M. pneumoniae* is known to induce ROS production and reduce glutathione levels in lung and lung carcinoma cells [3,40]. Furthermore, *M. pneumoniae* can inhibit host cell catalase, which could result in the toxicity of hydrogen peroxide in skin fibroblast and ciliated epithelial cells [41]. Together, these results implicate that the enhanced ROS production should be recognized as an important mechanism in the pathogenesis of *M. pneumoniae* infection [3].

In addition, many identified proteins were involved in extracellular matrix formation (Figure 4 and see Additional file 7: Figure S4A). Extracellular matrix plays an important role in regulating many cellular functions like adhesion, cell shape, migration, proliferation, polarity, differentiation, and apoptosis [42]. For example, SERPINE1, as a multifaceted proteolytic factor, not only functions as an inhibitor of the serine protease, but also plays an important role in signal transduction, cell adhesion, and migration [43]. Similarly, ADAM9, a member of the ADAM family, is involved in the proteolytic processing of multiple transmembrane proteins, as well as cell adhesion, migration, and signal transduction [44]. Gal-1 also displays diverse biological activities including cell adhesion, B cell development, mRNA splicing, angiogenesis and tissue differential/homeostasis, and inflammation [45]. Thus, targeting the interplay between host cells and microenviroment might be another important mechanism for *M. pneumoniae* pathogenesis.

Finally, we were interested in the potential clinical application of such secretomic study, e.g. biomarker or therapeutic target discovery [15]. To do that, we chose one of the identified proteins, IL-33, and conducted a “proof-of-concept” experiment. IL-33, a crucial amplifier of the innate immunity in infectious diseases as well as in autoimmune processes, is also a recently identified DAMP [46-48]. It has been shown that IL-33 plays an important role in driving antiviral CD8+ T cell responses in lymphocytic choriomeningitis virus-infected mice [47]. During the experimental intestinal nematodes (*Trichuris muris*) infection in mice, IL-33 was markedly elevated soon after infection [49]. Schmitz and co-workers demonstrated that injection of IL-33 into mice induced a profound eosinophilia with associated pathologic changes [50], and had potent effects on eosinophil, including the induced production of superoxide anion and IL-8, degranulation and eosinophil survival [51]. We found *M. pneumoniae* significantly increased IL-33 production in A549 cells, and IL-33 levels were significantly higher in MPP patients, implying an important role for IL-33 in *M. pneumoniae*-elicited immune response (Figure 7). Further ROC analysis revealed that IL-33 could help distinguish MPP patients from patients with foreign objects. Thus, manipulation of IL-33 might represent a promising new therapeutic strategy for treating the inflammatory disorder during *M. pneumoniae* infection.

## Conclusions

In the current study, we identified many differentially expressed secretory proteins during *M. pneumoniae* infection using the quantitative label-free MS method, through which complex regulatory networks have been revealed. Some of the proteins could be used as lead candidates for further functional and preclinical evaluation for their roles in *M. pneumoniae* infection. Such information will shed new light into the study of host response during *M. pneumoniae* infection for better understanding the underlying molecular mechanisms.

## Methods

### *Mycoplasma pneumoniae* culture

*M. pneumoniae* strain 29342 (American Type Culture Collection, Rockville, MD) was cultured in mycoplasma broth at 37°C under 5% (v/v) humidified CO_2_, consisting of mycoplasma broth base CM403 (OXIOD, Hampshire, United Kingdom), mycoplasma selective supplement G SR59 (OXIOD), 0.5% glucose, and 0.002% phenol red. Agar plates used for colony counting were prepared similarly, but containing mycoplasma agar base CM401 (OXIOD) instead of mycoplasma broth base CM403. The concentration of *M. pneumoniae* was quantified by measuring colony forming units (CFU).

### Cell cultures and preparation of conditioned media

As human alveolar epithelial carcinoma A549 cells (CCL-185, ATCC) are very tolerant to SFM, we chose them as a cell model for our secretome study [15]. Firstly, A549 cells were maintained in phenol red-free Dulbecco’s Modified Eagle Medium Nutrient Mixture (DMEM/F12) (Gibco, Grand Island, NY) supplemented with 10% fetal bovine serum (FBS) (Gibco) at 37°C in a humidified atmosphere containing 5% CO_2_. When A549 cells were grown to approximately 60-70% confluence, they were washed five times with SFM to remove albumin and other elements contained in FBS. Cells were then either infected with 10 CFU/cell of *M. pneumoniae* in SFM or left untreated for further conditioned media (CM) collection. Cell viability in SFM was assessed by MTT test and trypan blue exclusion assay, and the cell death was assessed by apoptosis assay using the Annexin V-FITC/PI Kit (Multiscience, Hangzhou, China).

### Sample preparation

The CM was harvested 24 h after infection by centrifugation at 9,000 g for 15 min to remove floating cells and cellular debris, and filtered through a 0.22 μm filter (Chemicon, Millipore, MA, USA). After the addition of protease inhibitors (Inhibitor cocktail complete, Roche Diagnostics, Mannheim, Germany), the media was concentrated using the Amicon Ultra-15 (Millipore) centrifugal filter devices with a 3,000-nomina-weight limit (NMWL). The supernatants were subsequently precipitated by acetone at -20°C overnight, and harvested by centrifugation at 16,000 g for 20 min. The protein pellets were dried in air and then resuspended in an appropriate volume of reducing solution containing 6 M urea, 2 M thiourea and 25 mM ammonium bicarbonate (Sigma, St Louis, MO). The protein concentrations were determined by the Bradford assay (Bio-Rad, Hercules, CA). 100 μg of each sample was reduced with 10 mM DTT (Sigma) at 37°C for 2.5 h, and then carbamidomethylated with 50 mM iodoacetamide (IAA) (Sigma) at room temperature in the dark for 40 min. Subsequently, digestion was performed by sequencing grade trypsin (Promega, Madison, WI) using a 1:50 enzyme:protein ratio at 37°C for 20 h. After digestion, samples were lyophilized under vacuum and kept at -80°C until use. Three independent experiments were performed and samples were prepared individually for further study.

Total cell lysates from the A549 cells were prepared as previously described [3]. Briefly, cells were washed and detached on ice in phosphate-buffered saline (PBS), and lysed in cell lysis buffer containing 7 M urea, 2 M thiourea, 4% CHAPS, 65 mM DTT, and 0.2% biolyte (Bio-Rad). The lysates were frozen and thawed with liquid nitrogen three times, and then centrifuged for 1 h at 10,000 g to remove cellular debris. The supernatant was then collected for further Western blot analysis.

### LC-MS/MS

All of the mass analyses were performed using a nano-LC-MS/MS system, which consisted of a nano-HPLC system (the Ettan MDLC system; GE Healthcare, Piscataway, NJ) and a linear trap quadruple (LTQ) mass spectrometer (LTQ VELOS; Thermo Finnigan, San Jose, CA) equipped with a nano-ESI source. A RP trap column (Zorbax 300SB-C18 peptide traps, Agilent Technologies, Wilmington, DE) was used for desalting of samples, and a C18 reverse-phase column (150 μm i.d., 150 mm length, Column Technology Inc., Fremont, CA) was used for separation. Mobile phase A consisted of HPLC-grade water containing 0.1% formic acid (FA), and phase B consisted of 84% HPLC-grade acetonitrile (ACN) containing 0.1% FA. The analytical separation was run at a flow rate of 2 μl/min by using a linear gradient of phase B as follows: 4%-50% for 105 min, 50%-100% for 9 min and 100% for 6 min. The eluent was then introduced into the LTQ mass spectrometer with the ESI spray voltage set at 3.2 kV. For MS survey scans, each scan cycle consisted of one full MS scan, and five MS/MS events were analyzed. The LC-MS/MS analyses were repeated three times for each independent biological sample. Then the LC-MS/MS results were pooled for each biological replicates to reduce technical variation.

### Data analysis and label-free quantitation

We created the peak lists from original RAW files with Bioworks Browser software (version 3.1, Thermo Electron, San Jose, CA) with the minimum peak intensity of 1000. Peptide identification was performed from each experiment with TurboSEQUEST program in the Bioworks Browser software suite by automatically searching against the nonredundant International Protein Index (IPI) human protein database (version 3.60) with decoy sequences (reverse of target database). The search parameters were set as: (a) trypsin digestion; (b) up to two missed cuts allowed; (c) cysteine carbamidomethylation as a fixed modification and methionine oxidation as a variable modification; and (d) mass tolerances set at 3.0 Da for the precursor ions and 1.0 Da for fragment ones. For protein identification, Delta Cn (≥0.1) and cross-correlation scores (Xcorr, one charge ≥1.9, two charges ≥2.2, three charges ≥3.75) were required. Only proteins identified by at least two unique peptides with good-quality tandem MS/MS data were reported. False discovery rate (FDR) was calculated by searching against a sequence-reversed decoy IPI human version 3.60 databases using the same search parameters and was estimated to be 2.0%. Multiple or ambiguous IDs were not allowed, and the decoy database hits were removed from the results. We also removed frequently observed contaminants such as porcine trypsin and human keratins.

To estimate the fold-changes in the levels of identified proteins between the experimental groups, we used DeCyder MS Differential Analysis Software (DeCyder MS, version 2.0, GE Healthcare) for comparison and label-free relative quantitation of LC-MS/MS data [52,53]. The relative quantitation analysis consisted of two main procedures. Firstly, the PepDetect module of the software was employed for automated peptide detection, charge state assignments based on resolved isotopic peaks and consistent spacing between consecutive charge states, and quantitation based on MS signal intensities. The final step was to automatically match peptide within a mass and time tolerance window (0.5 Da and 2 min, respectively) across different signal intensity maps with PepMatch module, resulted in a quantitative comparison. The entire peptide intensity distributions detected in each sample were applied for normalization and peptides were identified by importing TurboSEQUEST search results to the PepMatch module.

### Bioinformatics analysis

Classical secretory proteins with a signal peptide were predicted by SignalP4.1 and were selected on the basis of their D-value above 0.45 [54]. Non-classical secretory proteins without a signal peptide were predicted by SecretomP 2.0 and were selected by their neural network (NN) score≥0.5 [55]. Simultaneously, all the identified proteins were searched against ExoCarta data to determine whether they were present in exosome fractions [22]. The identified proteins were classified on the basis of their cellular compartment by Gene Ontology (GO) annotation [56]. The enrichment analysis of functional annotation clustering based on cellular compartment were performed by Database for Annotation, Visualization and Integrated Discovery (DAVID) Bioinformatics Resources 6.7, with an enrichment score≥1.3 and an EASE score < 0.05 [57]. DAVID 6.7 was also used to recognize functional Kyoto Encyclopedia of Genes and Genomes (KEGG) pathway categories [58]. Biological Networks Gene Ontology (BiNGO) (version 2.44), a Cytoscape plugin (version 2.8.2), was also used to determine over-representation of GO categories [59]. Over-representation statistics were calculated by means of hypergeometric analysis followed by Benjamini & Hochberg FDR correction. Finally, Search Tool for the Retrieval of Interacting Genes (STRING) 9.05 was performed to construct a network model showing protein interactions based on known and predicted protein-protein interactions [26].

### Western blotting

Western blots were performed as described previously, with some modifications [3]. Briefly, equal amounts of protein from total cell lysates or concentrated cell culture supernatants were denatured, separated on 12% SDS-PAGE gels and transferred to PVDF membranes (Millipore). For detection, the membranes were incubated with various primary antibodies overnight at 4°C, followed by addition of fluorescence-labeled secondary antibody (Li-COR Biosciences, diluted 1:5000) for 1 h at room temperature. The membranes were then scanned using the Odyssey infrared imaging system (LI-COR Bioscience). The primary antibodies utilized included rabbit polyclonal anti-ADAM9 antibody (Cell Signaling Technolgoy, Beverly, MA, USA, diluted 1:1000), rabbit polyclonal anti-Gal1 antibody (Proteintech, Chicago, IL, diluted 1:1500), rabbit polyclonal anti-MIF antibody (Proteintech, diluted 1:2000), rabbit polyclonal anti-IL33 antibody (Proteintech, diluted 1:600), rabbit polyclonal anti-SERPINE1 antibody (Proteintech, diluted 1:800), rabbit polyclonal anti-IGFBP4 antibody (Millipore, diluted 1:1000), mouse monoclonal anti-β-actin antibody (Upstate, Lake Placid, NY, diluted 1:3000).

### Quantitative real-time PCR

Total RNA was extracted using TRIzol reagent (Invitrogen, Carlbad, CA) according to the manufacturer’s instructions. Subsequently, 500 ng of the RNA was reverse transcribed into cDNA using PrimeScript RT reagent Kit (Takara, Otsu, Japan), and then quantitative real-time PCR was carried out in an ABI PRISM 7900HT Real-Time PCR system using the SYBR Premix Ex Taq Kit (Takara) according to the manufacturer’s instructions. The thermal cycling conditions were: 30 sec at 95°C for initial denaturation, followed by 40 cycles of 5 sec at 95°C, 30 sec at 60°C for amplification, and 15 sec at 95°C, 1 min at 60°C and 15 sec at 95°C for melting curve analysis. Target gene primers are presented in Additional file 8: Table S3, in the supplemental material. An untreated cell sample was used as the calibrator and the fold-change for this sample was set as 1. Target gene Ct values were normalized to β-actin, and the results were analyzed by means of the 2^-△△Ct^ method [60].

### Measurement of IL-33 cytokine by enzyme linked immunosorbent assay

Peripheral blood and bronchoalveolar lavage fluid (BALF) samples of 30 pediatric patients with MPP (aged from 2.08-8.75 years old) were collected from Children’s Hospital, Zhejiang University School of Medicine from January 2012 to December 2012. Samples from age-matched children (aged from 2.50-8.50 years old) with foreign body in bronchus were used as controls. All samples were collected with informed consent from their guardians. This study was approved by the Ethics Committee of the Children’s Hospital, Zhejiang University School of Medicine. The procedure of fiberoptic bronchoscopy (FOB) and BALF collection were performed as described previously [61]. The samples were centrifuged at 2000 g for 10 min, and the supernatants were stored at -80°C until analysis. The levels of IL-33 in serum and BALF were determined using the IL-33 enzyme-linked immunosorbent assay (ELISA) kits (R&D Systems, Minneapolis, MN, USA) according to the manufacturer’s protocol.

### Statistical analysis

Each experiment was repeated at least three times independently. Data were expressed as mean ± SD and evaluated with Student’s t-test or Mann–Whitney U test. *p* < 0.05 was considered statistically significant.

## Competing interests

The authors declare that they have no competing interest.

## Authors’ contributions

ZMC and JY created the concept and design of this study. SXL and XJL performed the experiments. YSW participated in sample diagnosis and collection. SXL and YSW were responsible for the bioinformatic analysis and statistical analysis. SXL, ZMC and JY drafted, revised and edited the manuscript. SGS revised and edited the manuscript. All authors read and approved the final manuscript.

## Supplementary Material

Additional file 1: Figure S1 Assessment of A549 cell growth in serum-free medium. (A) Relative viability of cells was determined by the MTT assay. Mean absorption was normalized to control, with controls (untreated + SM group) being 100%. (B) Cell growth rate was investigated by cell count. (C) Cell viability was measured by Trypan blue exclusion assay. (D) Micrographs (200×) of cell morphology. The values represent averages of three independent experiments with six replicate detections (mean ± SD). *, *M. pneumoniae*-treated + SFM group vs *M. pneumoniae*-treated + SM group, *p*<0.05; #, Untreated + SFM group vs Untreated + SM group, *p*<0.05. SM: medium containing 10% FBS; SFM: serum-free medium. Click here for file

Additional file 2: Figure S2 Cell death analysis of A549 cells growing in SFM for 24 h. Cell apoptosis/necrosis was analyzed by dual-parameter flow cytometry stained with Annexin V-FITC and PI. (A) Representative dot plot images from three independent experiments. (B) Quantitative analysis results from (A). Data are presented as mean ± SD. Click here for file

Additional file 3: Figure S3 Venn diagrams of identified proteins. The overlaps of identified proteins in each biological replicate were shown in (A) for untreated and (B) for *M. pneumoniae*-treated A549 cells. (C) shows the overlaps of the non-redundant proteins identified between control and infected cells. Click here for file

Additional file 4: Datasheet S1 Database search results for all the secretory proteins identified in this study. Click here for file

Additional file 5: Table S1 Basic information of identified proteins. Click here for file

Additional file 6: Table S2 Differentially expressed proteins identified in the secretome of *Mycoplasma pneumoniae*-infected A549 and untreated A549 cells. Click here for file

Additional file 7: Figure S4 Functional gene ontology (GO) analysis of the differentially expressed secretory proteins during *M. pneumoniae* infection. (A) GO analysis of cellular component distribution for proteins that are down-regulated by *M. pneumoniae* treatment. (B) GO analysis of molecular function distribution for proteins that are up-regulated by *M. pneumoniae* treatment. (C) GO analysis of molecular function distribution for proteins that are down-regulated by *M. pneumoniae* treatment. (D) GO analysis of biological process distribution of clusters for proteins that are up-regulatedby *M. pneumoniae* treatment. (E) GO analysis of biological process distribution of clusters for proteins that are down-regulated by *M. pneumoniae* treatment. Over-representation of GO categories was analyzed using the Biological Networks Gene Ontology plugin (BINGO, version 2.44). Over-representation statistics were calculated by using the hypergeometric analysis and Benjamini & Hochberg False Discovery Rate (FDR) correction. Only categories that are significantly enriched after correction are represented. The color scales indicate the *p* value range for over-representation. The node size is proportional to the number of proteins annotated with the GO term. Click here for file

Additional file 8: Table S3 Primers used for PCR amplification. Click here for file
