# Supplementary figures and images for: Global secretome characterization of A549 human alveolar epithelial carcinoma cells during Mycoplasma pneumoniae infection
Source: BMC Microbiol. 2014 Feb 7;14:27. doi: 10.1186/1471-2180-14-27 (PMC3922035; doi:10.1186/1471-2180-14-27)

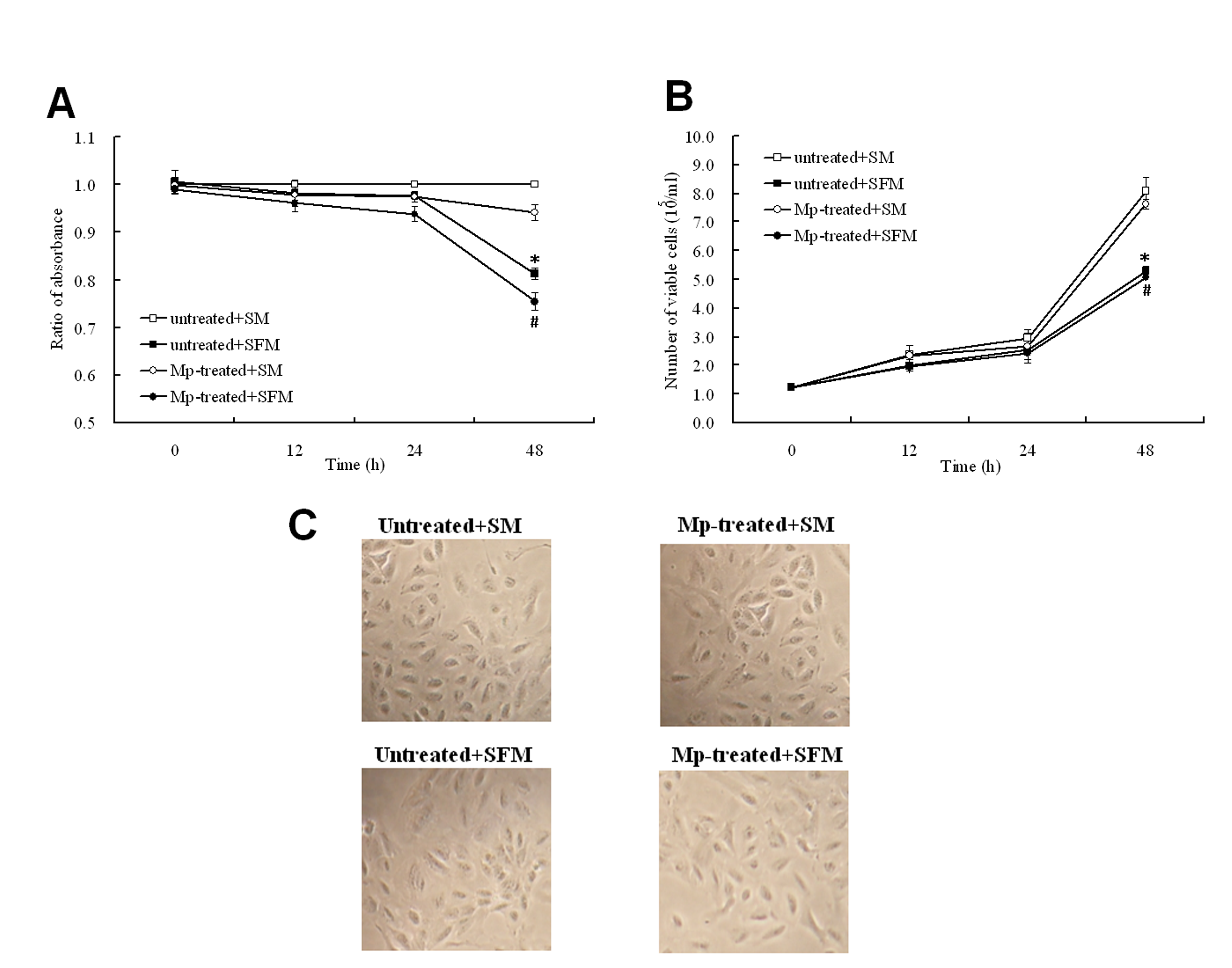

Supplement: Additional file 1: Figure S1 — Assessment of A549 cell growth in serum-free medium. (A) Relative viability of cells was determined by the MTT assay. Mean absorption was normalized to control, with controls (untreated + SM group) being 100%. (B) Cell growth rate was investigated by cell count. (C) Cell viability was measured by Trypan blue exclusion assay. (D) Micrographs (200×) of cell morphology. The values represent averages of three independent experiments with six replicate detections (mean ± SD). *, M. pneumoniae-treated + SFM group vs M. pneumoniae-treated + SM group, p<0.05; #, Untreated + SFM group vs Untreated + SM group, p<0.05. SM: medium containing 10% FBS; SFM: serum-free medium. [file 1471-2180-14-27-S1.tiff]

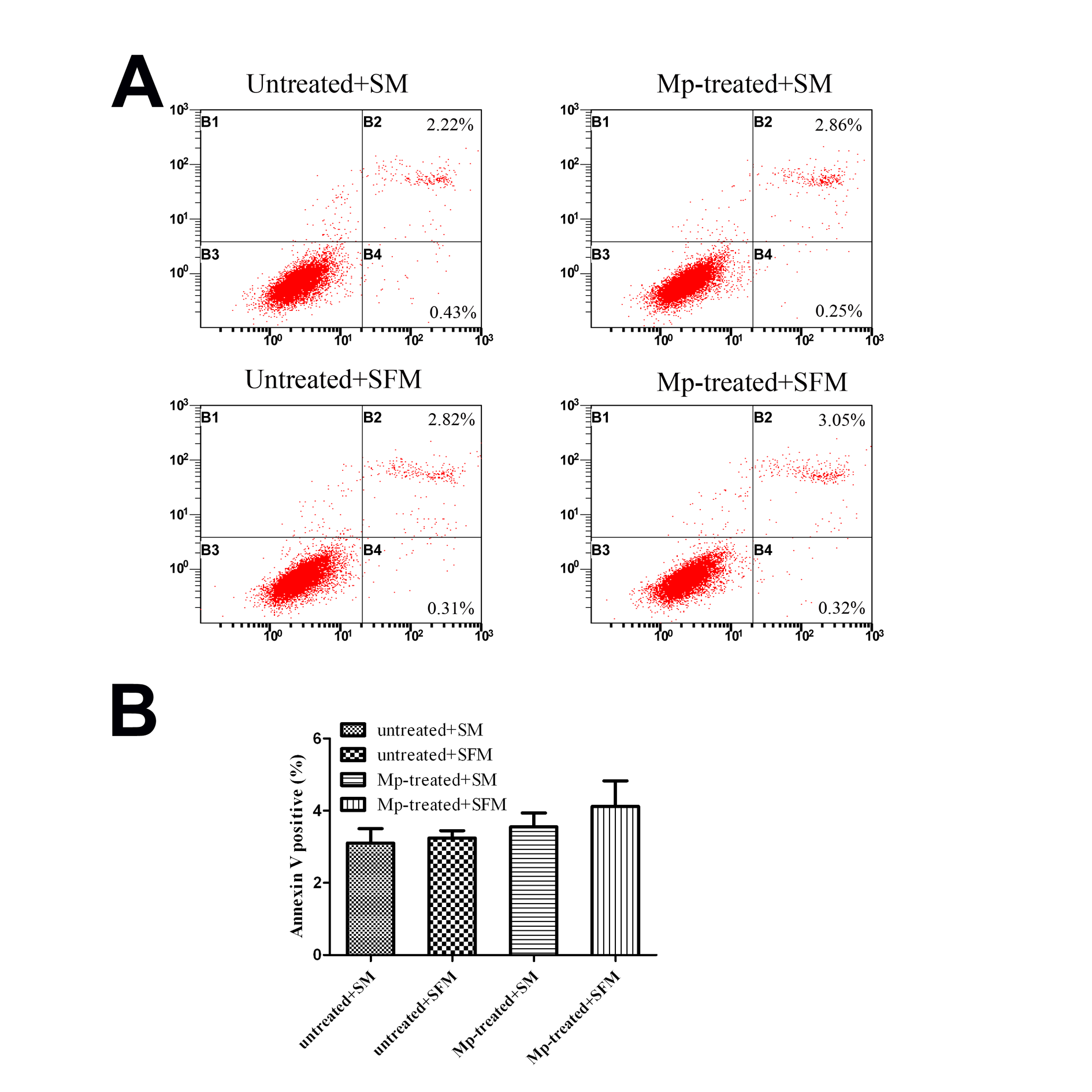

Supplement: Additional file 2: Figure S2 — Cell death analysis of A549 cells growing in SFM for 24 h. Cell apoptosis/necrosis was analyzed by dual-parameter flow cytometry stained with Annexin V-FITC and PI. (A) Representative dot plot images from three independent experiments. (B) Quantitative analysis results from (A). Data are presented as mean ± SD. [file 1471-2180-14-27-S2.tiff]

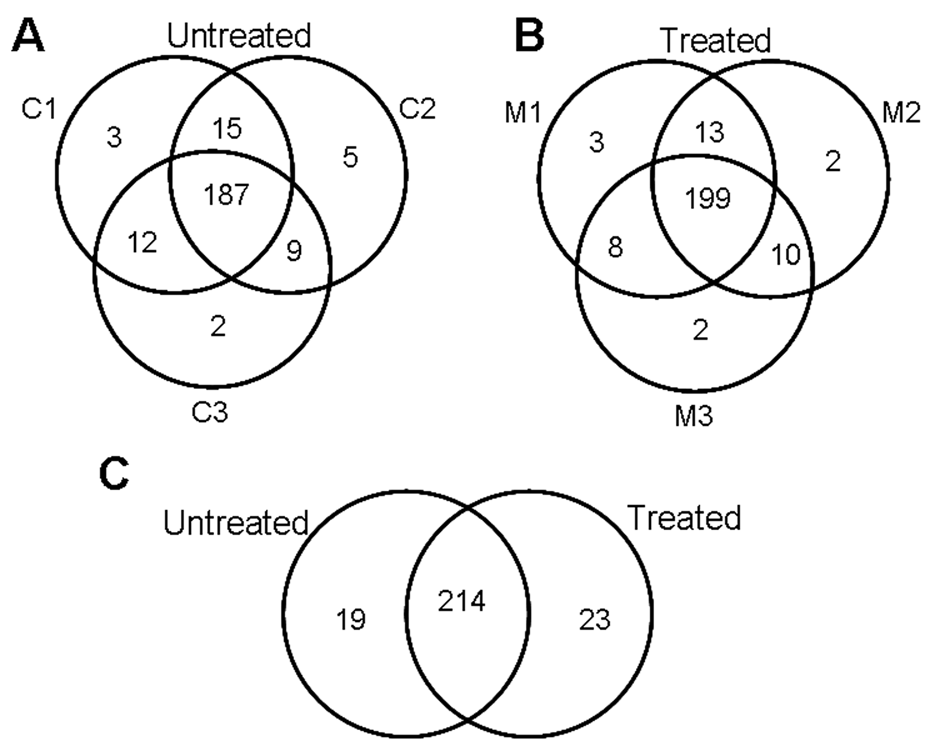

Supplement: Additional file 3: Figure S3 — Venn diagrams of identified proteins. The overlaps of identified proteins in each biological replicate were shown in (A) for untreated and (B) for M. pneumoniae-treated A549 cells. (C) shows the overlaps of the non-redundant proteins identified between control and infected cells. [file 1471-2180-14-27-S3.tiff]

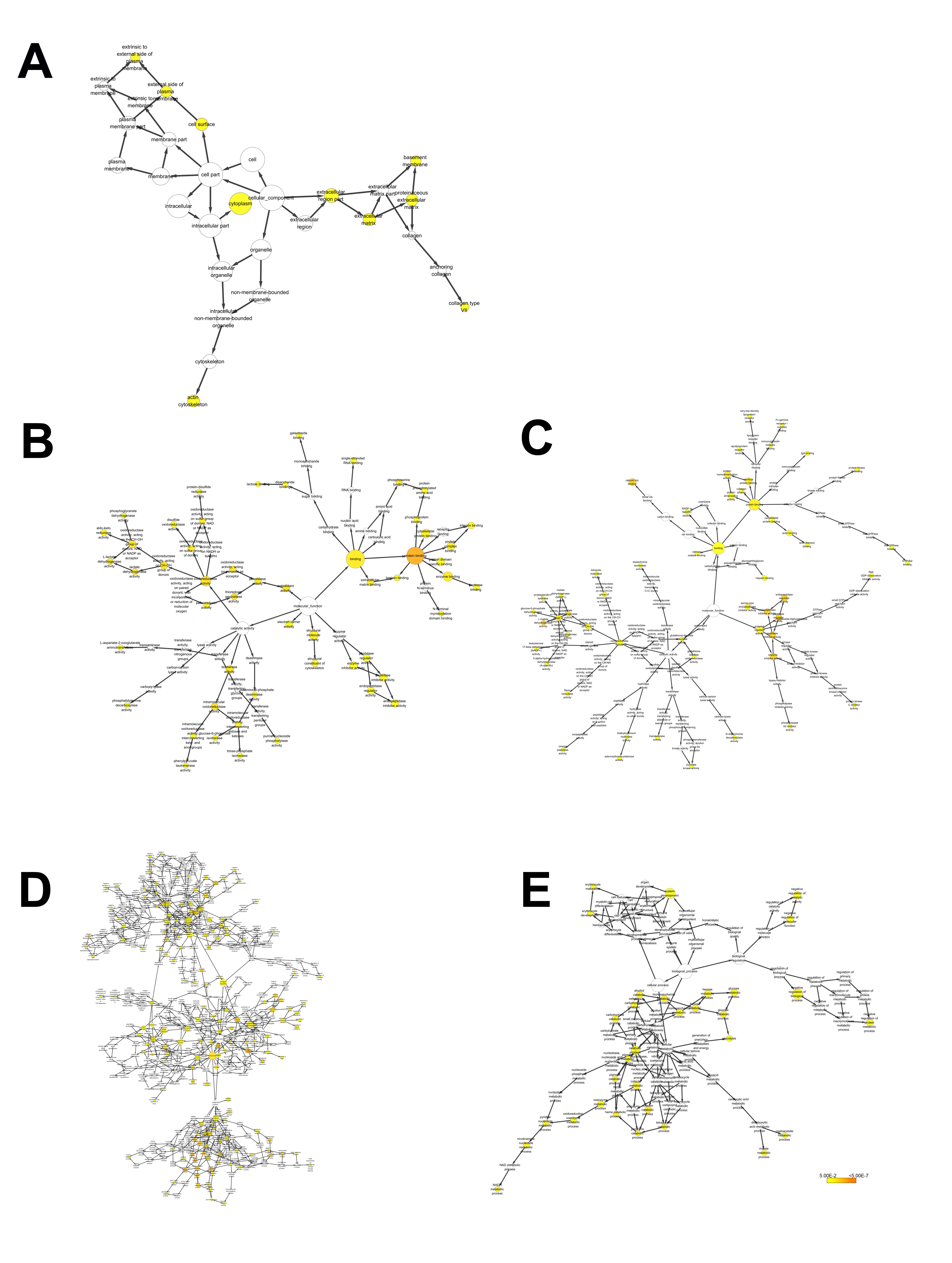

Supplement: Additional file 7: Figure S4 — Functional gene ontology (GO) analysis of the differentially expressed secretory proteins during M. pneumoniae infection. (A) GO analysis of cellular component distribution for proteins that are down-regulated by M. pneumoniae treatment. (B) GO analysis of molecular function distribution for proteins that are up-regulated by M. pneumoniae treatment. (C) GO analysis of molecular function distribution for proteins that are down-regulated by M. pneumoniae treatment. (D) GO analysis of biological process distribution of clusters for proteins that are up-regulatedby M. pneumoniae treatment. (E) GO analysis of biological process distribution of clusters for proteins that are down-regulated by M. pneumoniae treatment. Over-representation of GO categories was analyzed using the Biological Networks Gene Ontology plugin (BINGO, version 2.44). Over-representation statistics were calculated by using the hypergeometric analysis and Benjamini & Hochberg False Discovery Rate (FDR) correction. Only categories that are significantly enriched after correction are represented. The color scales indicate the p value range for over-representation. The node size is proportional to the number of proteins annotated with the GO term. [file 1471-2180-14-27-S7.tiff]
