# Supplementary material for: Global secretome characterization of A549 human alveolar epithelial carcinoma cells during Mycoplasma pneumoniae infection
Source: BMC Microbiol. 2014 Feb 7;14:27. doi: 10.1186/1471-2180-14-27 (PMC3922035; doi:10.1186/1471-2180-14-27)
Supplement: Additional file 5: Table S1 — Basic information of identified proteins. [file 1471-2180-14-27-S5.doc]

| **Table S1. Basic information of identified proteins** | | | | | | | | |  |
| --- | --- | --- | --- | --- | --- | --- | --- | --- | --- |
| IPI | Protein name | Origin (A549, Mp-infected A549, both) | SWISS-PROT | Gene name | GENE ID | Position | SignalP | SecP score | ExoCarta |
| IPI00218914.5 | Retinal dehydrogenase 1 | both | P00352 | ALDH1A1 | 216 | 9q21.13 | No | 0.501 | ExoCarta_216 |
| IPI00018219.1 | Transforming growth factor-beta-induced protein ig-h3 | both | Q15582 | TGFBI | 7045 | 5q31 | Yes | 0.454 | ExoCarta_7045 |
| IPI00465248.5 | Isoform alpha-enolase of Alpha-enolase | both | P06733 | ENO1 | 2023 | 1p36.2 | No | 0.536 | ExoCarta_2023 |
| IPI00105407.1 | Aldo-keto reductase family 1 member B10 | both | O60218 | AKR1B10 | 57016 | 7q33 | No | 0.338 | ExoCarta_57016 |
| IPI00295542.5 | Nucleobindin-1 | both | Q02818 | NUCB1 | 4924 | 19q13.33 | Yes | 0.305 | ExoCarta_4924 |
| IPI00003865.1 | Isoform 1 of Heat shock cognate 71 kDa protein | both | P11142 | HSPA8 | 3312 | 11q24.1 | No | 0.229 | ExoCarta_3312 |
| IPI00302592.2 | Isoform 2 of Filamin-A | both | P21333 | FLNA | 2316 | Xq28 | No | 0.446 | ExoCarta_2316 |
| IPI00291262.3 | Clusterin | both | P10909 | CLU | 1191 | 8p21-p12 | Yes | 0.826 | ExoCarta_1191 |
| IPI00418471.6 | Vimentin | both | P08670 | VIM | 7431 | 10p13 | No | 0.512 | ExoCarta_7431 |
| IPI00797270.4 | RCTPI1 Isoform 1 of Triosephosphate isomerase | both | P60174 | TPI1 | 7167 | 12p13 | No | 0.510 | ExoCarta_7167 |
| IPI00930688.1 | Tubulin alpha-1B chain | both | P68363 | TUBA1B | 10376 | 12q13.12 | No | 0.472 | ExoCarta_10376 |
| IPI00479186.7 | Isoform M2 of Pyruvate kinase isozymes M1/M2 | both | P14618 | PKM2 | 5315 | 15q22 | No | 0.420 | ExoCarta_5315 |
| IPI00013808.1 | Alpha-actinin-4 | both | O43707 | ACTN4 | 81 | 19q13 | No | 0.418 | ExoCarta_81 |
| IPI00029723.1 | Follistatin-related protein 1 | both | Q12841 | FSTL1 | 11167 | 3q13.33 | Yes | 0.533 | No results found |
| IPI00021439.1 | Actin, cytoplasmic 1 | both | P60709 | ACTB | 60 | 7p22 | No | 0.498 | ExoCarta_60 |
| IPI00218343.4 | Tubulin alpha-1C chain | both | Q9BQE3 | TUBA1C | 84790 | 12q13.12 | No | 0.481 | ExoCarta_84790 |
| IPI00180675.4 | Tubulin alpha-1A chain | M | Q71U36 | TUBA1A | 7846 | 12q13.12 | No | 0.469 | ExoCarta_7846 |
| IPI00643920.3 | cDNA FLJ54957, highly similar to Transketolase | both | P29401 | TKT | 7086 | 3p14.3 | No | 0.315 | ExoCarta_7086 |
| IPI00007118.1 | Plasminogen activator inhibitor 1 | both | P05121 | SERPINE1 | 5054 | 7q22.1 | Yes | 0.644 | ExoCarta_5054 |
| IPI00291483.3 | Aldo-keto reductase family 1 member C3 | both | P42330 | AKR1C3 | 8644 | 10p15-p14 | No | 0.709 | No results found |
| IPI00007750.1 | Tubulin alpha-4A chain | M | P68366 | TUBA4A | 7277 | 2q35 | No | 0.550 | ExoCarta_7277 |
| IPI00021263.3 | 14-3-3 protein zeta/delta | both | P63104 | YWHAZ | 7534 | 8q23.1 | No | 0.252 | ExoCarta_7534 |
| IPI00554788.5 | Keratin, type I cytoskeletal 18 | both | P05783 | KRT18 | 3875 | 12q13 | No | 0.725 | ExoCarta_3875 |
| IPI00012011.6 | Cofilin-1 | both | P23528 | CFL1 | 1072 | 11q13 | No | 0.628 | ExoCarta_1072 |
| IPI00413641.7 | Aldose reductase | both | P15121 | AKR1B1 | 231 | 7q35 | Yes | 0.395 | ExoCarta_231 |
| IPI00000874.1 | Peroxiredoxin-1 | both | Q06830 | PRDX1 | 5052 | 1p34.1 | No | 0.528 | ExoCarta_5052 |
| IPI00465439.5 | Fructose-bisphosphate aldolase A | both | P04075 | ALDOA | 226 | 16p11.2 | No | 0.356 | ExoCarta_226 |
| IPI00022418.1 | Isoform 1 of Fibronectin | both | P02751 | FN1 | 2335 | 2q34 | Yes | 0.369 | ExoCarta_2335 |
| IPI00169383.3 | Phosphoglycerate kinase 1 | both | P00558 | PGK1 | 5230 | Xq13.3 | No | 0.389 | ExoCarta_5230 |
| IPI00219217.3 | L-lactate dehydrogenase B chain | both | P07195 | LDHB | 3945 | 12p12.2-p12.1 | No | 0.569 | ExoCarta_3945 |
| IPI00219446.5 | Phosphatidylethanolamine-binding protein 1 | both | P30086 | PEBP1 | 5037 | 12q24.23 | No | 0.672 | ExoCarta_5037 |
| IPI00216008.4 | Isoform Long of Glucose-6-phosphate 1-dehydrogenase | both | P11413 | G6PD | 2539 | Xq28 | No | 0.449 | ExoCarta_2539 |
| IPI00023673.1 | Galectin-3-binding protein | both | Q08380 | LGALS3BP | 3959 | 17q25 | Yes | 0.738 | ExoCarta_3959 |
| IPI00219018.7 | Glyceraldehyde-3-phosphate dehydrogenase | both | P04406 | GAPDH | 2597 | 12p13 | No | 0.467 | ExoCarta_2597 |
| IPI00910870.1 | cDNA FLJ59163, highly similar to Heat shock cognate 71 kDa protein | C | B4DTX2 | HSPA8 | - | - | No | 0.303 | No results found |
| IPI00554648.3 | Keratin, type II cytoskeletal 8 | both | P05787 | KRT8 | 3856 | 12q13 | No | 0.129 | ExoCarta_3856 |
| IPI00843975.1 | Ezrin | both | P15311 | EZR | 7430 | 6q25.3 | No | 0.563 | ExoCarta_7430 |
| IPI00022774.3 | Transitional endoplasmic reticulum ATPase | both | P55072 | VCP | 7415 | 9p13.3 | No | 0.163 | ExoCarta_7415 |
| IPI00021405.3 | Isoform A of Lamin-A/C | both | P02545 | LMNA | 4000 | 1q22 | No | 0.077 | ExoCarta_4000 |
| IPI00220642.7 | 14-3-3 protein gamma | both | P61981 | YWHAG | 7532 | 7q11.23 | No | 0.290 | ExoCarta_7532 |
| IPI00018146.1 | 14-3-3 protein theta | both | P27348 | YWHAQ | 10971 | 2p25.1 | No | 0.256 | ExoCarta_10971 |
| IPI00032292.1 | Metalloproteinase inhibitor 1 | both | P01033 | TIMP1 | 7076 | Xp11.3-p11.23 | Yes | 0.765 | ExoCarta_7076 |
| IPI00646909.2 | Tubulin alpha-8 chain | M | Q9NY65 | TUBA8 | 51807 | 22q11.1 | No | 0.568 | ExoCarta_51807 |
| IPI00216318.5 | Isoform Long of 14-3-3 protein beta/alpha | both | P31946 | YWHAB | 7529 | 20q13.1 | No | 0.352 | ExoCarta_7529 |
| IPI00217966.8 | L-lactate dehydrogenase | both | P00338 | LDHA | 3939 | 11p15.4 | No | 0.549 | ExoCarta_3939 |
| IPI00550363.3 | Transgelin-2 | both | P37802 | TAGLN2 | 8407 | 1q21-q25 | No | 0.784 | ExoCarta_8407 |
| IPI00550069.3 | Ribonuclease inhibitor | both | P13489 | RNH1 | 6050 | 11p15.5 | No | 0.568 | ExoCarta_6050 |
| IPI00179964.5 | Isoform 1 of Polypyrimidine tract-binding protein 1 | both | P26599 | PTBP1 | 5725 | 19p13.3 | No | 0.415 | ExoCarta_5725 |
| IPI00029733.1 | Aldo-keto reductase family 1 member C1 | both | Q04828 | AKR1C1 | 1645 | 10p15-p14 | No | 0.461 | No results found |
| IPI01019113.2 | Tubulin beta chain | M | P07437 | TUBB | 203068 | 6p21.33 | No | 0.494 | ExoCarta_203068 |
| IPI00939442.1 | Heat shock 70 kDa protein 1L | both | P34931 | HSPA1L | 3305 | 6p21.3 | No | 0.370 | ExoCarta_3305 |
| IPI00017696.1 | Complement C1s subcomponent | both | P09871 | C1S | 716 | 12p13 | Yes | 0.730 | ExoCarta_716 |
| IPI00003590.2 | Isoform 1 of Sulfhydryl oxidase 1 | both | O00391 | QSOX1 | 5768 | 1q24 | Yes | 0.611 | ExoCarta_5768 |
| IPI00186290.6 | Elongation factor 2 | both | P13639 | EEF2 | 1938 | 19p13.3 | No | 0.380 | ExoCarta_1938 |
| IPI00396378.3 | Isoform B1 of Heterogeneous nuclear | both | P22626 | HNRNPA2B1 | 3181 | 7p15 | No | 0.081 | ExoCarta_3181 |
| IPI00306959.10 | Keratin, type II cytoskeletal 7 | both | P08729 | KRT7 | 3855 | 12q13.13 | No | 0.188 | ExoCarta_3855 |
| IPI00216691.5 | Profilin-1 | both | P07737 | PFN1 | 5216 | 17p13.3 | No | 0.469 | ExoCarta_5216 |
| IPI00219757.13 | Glutathione S-transferase P | both | P09211 | GSTP1 | 2950 | 11q13 | No | 0.545 | ExoCarta_2950 |
| IPI00216049.1 | Isoform 1 of Heterogeneous nuclear ribonucleoprotein K | both | P61978 | HNRNPK | 3190 | 9q21.32-q21.33 | No | 0.177 | ExoCarta_3190 |
| IPI00016915.1 | Insulin-like growth factor-binding protein 7 | both | Q16270 | IGFBP7 | 3490 | 4q12 | Yes | 0.536 | ExoCarta_3490 |
| IPI00215965.2 | Isoform A1-B of Heterogeneous nuclear ribonucleoprotein A1 | both | P09651 | HNRNPA1 | 3178 | 12q13.1 | No | 0.106 | ExoCarta_3178 |
| IPI00925567.1 | Aldo-keto reductase family 1 member B15 | both | C9JRZ8 | AKR1B15 | 441282 | 7q33 | No | 0.387 | No results found |
| IPI00305380.3 | Insulin-like growth factor-binding protein 4 | both | P22692 | IGFBP4 | 3487 | 17q12-q21.1 | Yes | 0.887 | ExoCarta_3487 |
| IPI00018352.1 | Ubiquitin carboxyl-terminal hydrolase isozyme L1 | both | P09936 | UCHL1 | 7345 | 4p14 | No | 0.530 | No results found |
| IPI00003362.2 | HSPA5 protein | both | P11021 | HSPA5 | 3309 | 9q33.3 | Yes | 0.745 | ExoCarta_3309 |
| IPI00295386.7 | Carbonyl reductase [NADPH] 1 | M | P16152 | CBR1 | 873 | 21q22.13 | No | 0.633 | ExoCarta_873 |
| IPI00027497.5 | Glucose-6-phosphate isomerase | both | P06744 | GPI | 2821 | 19q13.1 | No | 0.453 | ExoCarta_2821 |
| IPI00219365.3 | Moesin | both | P26038 | MSN | 4478 | Xq11.1 | No | 0.530 | ExoCarta_4478 |
| IPI00339269.1 | Heat shock 70 kDa protein 6 | M | P17066 | HSPA6 | 3310 | 1q23 | No | 0.269 | ExoCarta_3310 |
| IPI00024284.5 | Basement membrane-specific heparan sulfate proteoglycan core protein | both | P98160 | HSPG2 | 3339 | 1p36.1-p34 | Yes | - | ExoCarta_3339 |
| IPI00016862.1 | Isoform Mitochondrial of Glutathione reductase, mitochondrial | both | P00390 | GSR | 2936 | 8p21.1 | No | 0.540 | ExoCarta_2936 |
| IPI00032293.1 | Cystatin-C | both | P01034 | CST3 | 1471 | 20p11.21 | Yes | 0.937 | ExoCarta_1471 |
| IPI00216171.3 | Gamma-enolase | both | P09104 | ENO2 | 2026 | 12p13 | No | 0.599 | ExoCarta_2026 |
| IPI00419585.9 | Peptidyl-prolyl cis-trans isomerase A | both | P62937 | PPIA | 5478 | 7p13 | No | 0.339 | ExoCarta_5478 |
| IPI00291006.2 | Malate dehydrogenase, mitochondrial | both | P40926 | MDH2 | 4191 | 7cen-q22 | No | 0.644 | ExoCarta_4191 |
| IPI00012503.1 | Isoform Sap-mu-0 of Proactivator polypeptide | both | P07602 | PSAP | 5660 | 10q21-q22 | Yes | 0.785 | ExoCarta_5660 |
| IPI00795292.1 | NME2, Isoform 3 of Nucleoside diphosphate kinase B | both | P22392 | NME2 | 4831 | 17q21.3 | No | 0.330 | ExoCarta_4831 |
| IPI00549725.6 | Phosphoglycerate mutase 1 | both | P18669 | PGAM1 | 5223 | 10q25.3 | No | 0.407 | ExoCarta_5223 |
| IPI00026314.1 | Isoform 1 of Gelsolin | both | P06396 | GSN | 2934 | 9q33 | Yes | 0.553 | ExoCarta_2934 |
| IPI00000816.1 | 14-3-3 protein epsilon | both | P62258 | YWHAE | 7531 | 17p13.3 | No | 0.330 | ExoCarta_7531 |
| IPI00219525.10 | 6-phosphogluconate dehydrogenase, decarboxylating | M | P52209 | PGD | 5226 | 1p36.22 | No | 0.426 | ExoCarta_5226 |
| IPI00297284.1 | Insulin-like growth factor-binding protein 2 | both | P18065 | IGFBP2 | 3485 | 2q33-q34 | Yes | 0.886 | No results found |
| IPI00220301.5 | Peroxiredoxin-6 | both | P30041 | PRDX6 | 9588 | 1q25.1 | No | 0.378 | ExoCarta_9588 |
| IPI00787323.2 | Similar to Keratin, type II cytoskeletal 8 | M | - | - | - | - | No | 0.119 | No results found |
| IPI00017672.4 | cDNA FLJ25678 fis, clone TST04067, highly similar to PURINE NUCLEOSIDE PHOSPHORYLASE | M | P00491 | NP | 4860 | 14q13.1 | No | 0.509 | ExoCarta_4860 |
| IPI00003818.1 | Kynureninase | both | Q16719 | KYNU | 8942 | 2q22.2 | No | 0.426 | No results found |
| IPI00413344.3 | Cofilin-2 | both | Q9Y281 | CFL2 | 1073 | 14q12 | No | 0.683 | ExoCarta_1073 |
| IPI00023048.4 | Elongation factor 1-delta | both | P29692 | EEF1D | 1936 | 8q24.3 | No | 0.529 | ExoCarta_1936 |
| IPI00291136.4 | Collagen alpha-1(VI) chain | both | P12109 | COL6A1 | 1291 | 21q22.3 | Yes | 0.234 | ExoCarta_1291 |
| IPI00903145.1 | Radixin | both | P35241 | RDX | 5962 | 11q23 | No | 0.310 | ExoCarta_5962 |
| IPI00012007.6 | Adenosylhomocysteinase | both | P23526 | AHCY | 191 | 20q11.22 | No | 0.507 | ExoCarta_191 |
| IPI00009950.1 | Vesicular integral-membrane protein VIP36 | both | Q12907 | LMAN2 | 10960 | 5q35.3 | Yes | 0.222 | ExoCarta_10960 |
| IPI00513743.1 | Isoform 3 of Heat shock protein 105 kDa | both | Q92598 | HSPH1 | 10808 | 13q12.3 | No | 0.327 | ExoCarta_10808 |
| IPI00299024.9 | Brain acid soluble protein 1 | both | P80723 | BASP1 | 10409 | 5p15.1 | No | 0.470 | ExoCarta_10409 |
| IPI00289334.1 | Isoform 1 of Filamin-B | both | O75369 | FLNB | 2317 | 3p14.3 | No | 0.359 | ExoCarta_2317 |
| IPI00291175.7 | Isoform 1 of Vinculin | both | P18206 | VCL | 7414 | 10q22.2 | No | 0.181 | ExoCarta_7414 |
| IPI00014898.3 | Isoform 1 of Plectin-1 | both | Q15149 | PLEC1 | 5339 | 8q24 | No | - | No results found |
| IPI00303476.1 | ATP synthase subunit beta, mitochondrial | both | P06576 | ATP5B | 506 | 12q13.13 | No | 0.591 | ExoCarta_506 |
| IPI00027493.1 | 4F2 cell-surface antigen heavy chain | M | P08195 | SLC3A2 | 6520 | 11q13 | No | 0.644 | ExoCarta_6520 |
| IPI00216298.6 | Thioredoxin | both | P10599 | TXN | 7295 | 9q31 | No | 0.370 | ExoCarta_7295 |
| IPI00004656.3 | Beta-2-microglobulin | both | P61769 | B2M | 567 | 15q21-q22.2 | Yes | 0.907 | ExoCarta_567 |
| IPI00027851.2 | cDNA FLJ53927, highly similar to Beta-hexosaminidase alpha chain | both | P06865 | HEXA | 3073 | 15q24.1 | Yes | 0.701 | No results found |
| IPI00298547.3 | Protein DJ-1 | both | Q99497 | PARK7 | 11315 | 1p36.23 | No | 0.493 | ExoCarta_11315 |
| IPI00784119.1 | V-type proton ATPase subunit S1 | both | Q15904 | ATP6AP1 | 537 | Xq28 | Yes | 0.637 | ExoCarta_537 |
| IPI00296992.7 | Isoform Long of Tyrosine-protein kinase receptor UFO | both | P30530 | AXL | 558 | 19q13.1 | Yes | 0.363 | ExoCarta_558 |
| IPI00003919.1 | Glutaminyl-peptide cyclotransferase | both | Q16769 | QPCT | 25797 | 2p22.2 | Yes | 0.788 | Carta_25797 |
| IPI00027166.1 | Metalloproteinase inhibitor 2 | both | P16035 | TIMP2 | 7077 | 17q25 | Yes | 0.854 | ExoCarta_7077 |
| IPI00010796.1 | Protein disulfide-isomerase | M | P07237 | P4HB | 5034 | 17q25 | Yes | 0.679 | ExoCarta_5034 |
| IPI00215767.1 | Isoform Long of Beta-1,4-galactosyltransferase 1 | both | P15291 | B4GALT1 | 2683 | 9p13 | No | 0.732 |  |
| IPI00792115.1 | Putative uncharacterized protein DKFZp686H17246 | both | Q68DS3 | - | - | - | No | 0.503 | No results found |
| IPI00016610.2 | Poly(rC)-binding protein 1 | both | Q15365 | PCBP1 | 5093 | 2p13-p12 | No | 0.549 | ExoCarta_5093 |
| IPI00005668.4 | Aldo-keto reductase family 1 member C2 | C | P52895 | AKR1C2 | 1646 | 10p15-p14 | No | 0.452 | No results found |
| IPI00783862.2 | Flavin reductase | both | P30043 | BLVRB | 645 | 19q13.1-q13.2 | No | 0.834 | ExoCarta_645 |
| IPI00641368.4 | Tsukushin | both | Q8WUA8 | TSKU | 25987 | 11q13.5 | Yes | 0.724 | No results found |
| IPI00031420.3 | UDP-glucose 6-dehydrogenase | both | O60701 | UGDH | 7358 | 4p15.1 | No | 0.434 | ExoCarta_7358 |
| IPI00646304.4 | Peptidyl-prolyl cis-trans isomerase B | both | P23284 | PPIB | 5479 | 15q21-q22 | No | 0.853 | ExoCarta_5479 |
| IPI00784154.1 | 60 kDa heat shock protein, mitochondrial | both | P10809 | HSPD1 | 3329 | 2q33.1 | No | 0.289 | ExoCarta_3329 |
| IPI00022434.4 | Putative uncharacterized protein ALB | both | B2RBS8 | ALB | - | - | Yes | 0.468 | No results found |
| IPI00479722.2 | Proteasome activator complex subunit 1 | both | Q06323 | PSME1 | 5720 | 14q11.2 | No | 0.170 | ExoCarta_5720 |
| IPI00744692.1 | Transaldolase | both | P37837 | TALDO1 | 6888 | 11p15.5-p15.4 | No | 0.394 | ExoCarta_6888 |
| IPI00013890.2 | Isoform 1 of 14-3-3 protein sigma | both | P31947 | SFN | 2810 | 1p36.11 | No | 0.345 | ExoCarta_2810 |
| IPI00914848.1 | plasminogen activator inhibitor type 1, member 2 isoform b precursor | both | P07093 | SERPINE2 | 5270 | 2q36.1 | Yes | 0.692 |  |
| IPI00296183.7 | Aldehyde dehydrogenase, dimeric NADP-preferring | both | P30838 | ALDH3A1 | 218 | 17p11.2 | No | 0.686 | No results found |
| IPI00217481.3 | Isoform 1 of Probable G-protein coupled receptor 126 | both | Q86SQ4 | GPR126 | 57211 | 6q24.1 | Yes | 0.404 | No results found |
| IPI00473014.5 | Destrin | both | P60981 | DSTN | 11034 | 20p12.1 | No | 0.622 | ExoCarta_11034 |
| IPI00013894.1 | Stress-induced-phosphoprotein 1 | both | P31948 | STIP1 | 10963 | 11q13 | No | 0.344 | ExoCarta_10963 |
| IPI00023640.3 | Programmed cell death protein 5 | both | O14737 | PDCD5 | 9141 | 19q13.11 | No | 0.782 | ExoCarta_9141 |
| IPI00554786.5 | Isoform 5 of Thioredoxin reductase 1, cytoplasmic | both | Q16881 | TXNRD1 | 7296 | 12q23-q24.1 | No | 0.359 | No results found |
| IPI00292936.4 | C-X-C motif chemokine 5 | both | P42830 | CXCL5 | 6374 | 4q13.3 | Yes | 0.791 | No results found |
| IPI00940960.1 | cDNA FLJ59142, highly similar to Epididymal secretory protein E1 | both | P61916 | NPC2 | 10577 | 14q24.3 | Yes | 0.931 | No results found |
| IPI00171411.4 | Golgi membrane protein 1 | both | Q8NBJ4 | GOLM1 | 51280 | 9q21.33 | No | 0.547 | No results found |
| IPI00257882.7 | Xaa-Pro dipeptidase | M | P12955 | PEPD | 5184 | 19q13.11 | No | 0.479 | ExoCarta_5184 |
| IPI00028911.2 | Dystroglycan | both | Q14118 | DAG1 | 1605 | 3p21 | Yes | 0.110 | ExoCarta_1605 |
| IPI00163187.10 | Fascin | M | Q16658 | FSCN1 | 6624 | 7p22 | No | 0.385 | ExoCarta_6624 |
| IPI00299571.5 | Isoform 2 of Protein disulfide-isomerase A6 | M | Q15084 | PDIA6 | 10130 | 2p25.1 | Yes | 0.711 | ExoCarta_10130 |
| IPI00218918.5 | Annexin A1 | both | P04083 | ANXA1 | 301 | 9q21.13 | No | 0.511 | ExoCarta_301 |
| IPI00002280.1 | ProSAAS | both | Q9UHG2 | PCSK1N | 27344 | Xp11.23 | Yes | 0.707 | No results found |
| IPI00219861.3 | Isoform 1 of Low molecular weight phosphotyrosine protein phosphatase | C | P24666 | ACP1 | 52 | 2p25 | No | 0.903 | ExoCarta_52 |
| IPI00011229.1 | Cathepsin D | both | P07339 | CTSD | 1509 | 11p15.5 | Yes | 0.758 | ExoCarta_1509 |
| IPI00298994.6 | Talin-1 | both | Q9Y490 | TLN1 | 7094 | 9p13 | No | 0.233 | ExoCarta_7094 |
| IPI00029235.1 | Insulin-like growth factor-binding protein 6 | both | P24592 | IGFBP6 | 3489 | 12q13 | Yes | 0.675 | ExoCarta_3489 |
| IPI00013895.1 | Protein S100-A11 | both | P31949 | S100A11 | 6282 | 1q21 | No | 0.839 | ExoCarta_6282 |
| IPI00291005.8 | Malate dehydrogenase, cytoplasmic | both | P40925 | MDH1 | 4190 | 2p13.3 | No | 0.455 | ExoCarta_4190 |
| IPI00008529.1 | 60S acidic ribosomal protein P2 | both | P05387 | RPLP2 | 6181 | 11p15.5 | No | 0.265 | ExoCarta_6181 |
| IPI00025155.1 | Follistatin-related protein 3 | both | O95633 | FSTL3 | 10272 | 19p13 | Yes | 0.900 | No results found |
| IPI00007752.1 | Tubulin beta-2C chain | C | P68371 | TUBB2C | 10383 | 9q34 | No | 0.501 | No results found |
| IPI00179330.6 | UBB ubiquitin and ribosomal protein S27a precursor | both | P62979 | UBC;RPS27A; | 6233 | 2p16 | No | 0.880 | ExoCarta_6233 |
| IPI00013881.6 | Heterogeneous nuclear ribonucleoprotein H | both | P31943 | HNRNPH1 | 3187 | 5q35.3 | No | 0.254 | No results found |
| IPI00007797.3 | Fatty acid-binding protein, epidermal | both | Q01469 | FABP5 | 2171 | 8q21.13 | No | 0.363 | No results found |
| IPI00009997.1 | N-acetyllactosaminide beta-1,3-N-acetylglucosaminyltransferase | both | O43505 | B3GNT1 | 11041 | 11q13.2 | No | 0.914 | No results found |
| IPI00006114.4 | Pigment epithelium-derived factor | both | P36955 | SERPINF1 | 5176 | 17p13.3 | Yes | 0.825 | ExoCarta_5176 |
| IPI00335168.9 | MYL6B Isoform Non-muscle of Myosin light polypeptide 6 | both | P60660 | MYL6 | 4637 | 12q13.2 | No | 0.427 | ExoCarta_4637 |
| IPI00028109.1 | Protein dpy-30 homolog | C | Q9C005 | DPY30 | 84661 | 2p22.3 | No | 0.744 | No results found |
| IPI00219219.3 | Galectin-1 | both | P09382 | LGALS1 | 3956 | 22q13.1 | No | 0.345 | ExoCarta_3956 |
| IPI00797738.1 | Cytochrome c oxidase subunit 6B1 | both | P14854 | COX6B1 | 1340 | 19q13.1 | No | 0.511 | No results found |
| IPI00010896.3 | Chloride intracellular channel protein 1 | both | O00299 | CLIC1 | 1192 | 6p21.3 | No | 0.395 | ExoCarta_1192 |
| IPI00031086.1 | Insulin-like growth factor-binding protein 1 | both | P08833 | IGFBP1 | 3484 | 7p13-p12 | Yes | 0.826 | No results found |
| IPI00293303.1 | Legumain | M | Q99538 | LGMN | 5641 | 14q32.1 | Yes | 0.753 | No results found |
| IPI00376005.2 | Isoform 2 of Eukaryotic translation initiation factor 5A-1 | both | P63241 | EIF5A | 1984 | 17p13-p12 | No | 0.319 | ExoCarta_1984 |
| IPI00440932.1 | Isoform 1 of Disintegrin and metalloproteinase domain-containing protein 9 | both | Q13443 | ADAM9 | 8754 | 8p11.22 | Yes | 0.112 | ExoCarta_8754 |
| IPI00010706.1 | Glutathione synthetase | both | P48637 | GSS | 2937 | 20q11.2 | No | 0.484 | ExoCarta_2937 |
| IPI00953689.2 | Alpha-2-HS-glycoprotein | both | P02765 | AHSG | 197 | 3q27 | Yes | 0.536 | ExoCarta_197 |
| IPI00012048.1 | Isoform 1 of Nucleoside diphosphate kinase A | C | P15531 | NME1 | 4830 | 17q21.3 | No | 0.361 | ExoCarta_4830 |
| IPI00290462.5 | Carbonyl reductase [NADPH] 3 | both | O75828 | CBR3 | 874 | 21q22.2 | No | 0.674 | ExoCarta_874 |
| IPI00003881.5 | Heterogeneous nuclear ribonucleoprotein F | M | P52597 | HNRNPF | 3185 | 10q11.21 | No | 0.475 | ExoCarta_3185 |
| IPI00025512.2 | Heat shock protein beta-1 | both | P04792 | HSPB1 | 3315 | 7q11.23 | No | 0.740 | ExoCarta_3315 |
| IPI00645078.1 | Ubiquitin-like modifier-activating enzyme 1 | both | P22314 | UBA1 | 7317 | Xp11.23 | No | 0.530 | ExoCarta_7317 |
| IPI00646689.1 | Thioredoxin domain-containing protein 17 | both | Q9BRA2 | TXNDC17 | 84817 | 17p13.1 | No | 0.491 | ExoCarta_84817 |
| IPI00022426.1 | Protein AMBP | both | P02760 | AMBP | 259 | 9q32-q33 | Yes | 0.769 | ExoCarta_259 |
| IPI00000075.3 | Transforming growth factor beta-1 | both | P01137 | TGFB1 | 7040 | 19q13.1 | Yes | 0.868 | ExoCarta_7040 |
| IPI00002966.2 | Heat shock 70 kDa protein 4 | both | P34932 | HSPA4 | 3308 | 5q31.1 | No | 0.226 | ExoCarta_3308 |
| IPI00029658.1 | Isoform 1 of EGF-containing fibulin-like extracellular matrix protein 1 | both | Q12805 | EFEMP1 | 2202 | 2p16 | Yes | 0.719 | ExoCarta_2202 |
| IPI00296165.6 | cDNA FLJ54471, highly similar to Complement C1r subcomponent | both | P00736 | C1R | 715 | 12p13 | Yes | 0.697 | ExoCarta_715 |
| IPI00306322.2 | Collagen alpha-2(IV) chain | both | P08572 | COL4A2 | 1284 | 13q34 | Yes | 0.068 | ExoCarta_1284 |
| IPI00334587.1 | Isoform 2 of Heterogeneous nuclear ribonucleoprotein A/B | both | Q99729 | HNRNPAB | 3182 | 5q35.3 | No | 0.147 | No results found |
| IPI00844215.1 | Isoform 1 of Spectrin alpha chain, brain | both | Q13813 | SPTAN1 | 6709 | 9q34.11 | No | 0.237 | ExoCarta_6709 |
| IPI00385326.2 | Isoform 1 of Olfactomedin-like protein 2A | both | Q68BL7 | OLFML2A | 169611 | 9q33.3 | Yes | 0.467 | No results found |
| IPI00020956.1 | Hepatoma-derived growth factor | both | P51858 | HDGF | 3068 | 1q21-q23 | No | 0.477 | No results found |
| IPI00024175.3 | Isoform 1 of Proteasome subunit alpha type-7 | both | O14818 | PSMA7 | 5688 | 20q13.33 | No | 0.248 | ExoCarta_5688 |
| IPI00257508.4 | Dihydropyrimidinase-related protein 2 | both | Q16555 | DPYSL2 | 1808 | 8p22-p21 | No | 0.411 | ExoCarta_1808 |
| IPI00027463.1 | Protein S100-A6 | both | P06703 | S100A6 | 6277 | 1q21 | No | 0.577 | ExoCarta_6277 |
| IPI00007765.5 | Stress-70 protein, mitochondrial | both | P38646 | HSPA9 | 3313 | 5q31.1 | No | 0.280 | ExoCarta_3313 |
| IPI00006608.1 | Isoform APP770 of Amyloid beta A4 protein (Fragment) | M | P05067 | APP | 351 | 21q21.3 | Yes | 0.441 | ExoCarta_351 |
| IPI00010779.4 | Isoform 1 of Tropomyosin alpha-4 chain | M | P67936 | TPM4 | 7171 | 19p13.1 | No | 0.417 | ExoCarta_7171 |
| IPI00103175.1 | Isoform 1 of Soluble calcium-activated nucleotidase 1 | both | Q8WVQ1 | CANT1 | 124583 | 17q25.3 | No | 0.635 | No results found |
| IPI00553185.2 | T-complex protein 1 subunit gamma | both | P49368 | CCT3 | 7203 | 1q23 | No | 0.490 | ExoCarta_7203 |
| IPI00303882.2 | Isoform B of Mannose-6-phosphate receptor-binding protein 1 | both | O60664 | M6PRBP1 | 10226 | 19p13.3 | No | 0.559 | No results found |
| IPI00384051.6 | Putative uncharacterized protein PSME2 | both | Q9UL46 | PSME2 | 5721 | 14q11.2 | No | 0.363 | ExoCarta_5721 |
| IPI00553164.4 | RPSA 40S ribosomal protein SA | both | P08865 | RPSAP15 | 3921 | 3p22.2 | No | 0.620 | ExoCarta_3921 |
| IPI00015029.1 | Prostaglandin E synthase 3 | both | Q15185 | PTGES3 | 10728 | 12q13.3 | No | 0.729 | No results found |
| IPI00045396.1 | Isoform 2 of Calumenin | both | O43852 | CALU | 813 | 7q32.1 | Yes | 0.753 | ExoCarta_813 |
| IPI00375441.2 | Isoform 1 of Far upstream element-binding protein 1 | C | Q96AE4 | FUBP1 | 8880 | 1p31.1 | No | 0.111 | No results found |
| IPI00024915.2 | Isoform Mitochondrial of Peroxiredoxin-5, mitochondrial | both | P30044 | PRDX5 | 25824 | 11q13 | No | 0.703 | ExoCarta_25824 |
| IPI00024933.3 | Isoform 1 of 60S ribosomal protein L12 | both | P30050 | RPL12 | 6136 | 9q34 | No | 0.865 | ExoCarta_6136 |
| IPI00410714.5 | Hemoglobin subunit alpha | both | P69905 | HBA1 | 3039 | 16p13.3 | No | 0.349 | ExoCarta_3039 |
| IPI00000581.6 | cDNA FLJ56307, highly similar to Ubiquitin thioesterase protein OTUB1 | both | B4DPD5 | OTUB1 | - | - | No | 0.561 | ExoCarta_55611 |
| IPI00455315.4 | Annexin A2 | both | P07355 | ANXA2 | 302 | 15q22.2 | No | 0.746 | ExoCarta_302 |
| IPI00219029.3 | Aspartate aminotransferase, cytoplasmic | both | P17174 | GOT1 | 2805 | 10q24.1-q25.1 | No | 0.439 | ExoCarta_2805 |
| IPI00290085.2 | Cadherin-2 | both | P19022 | CDH2 | 1000 | 18q11.2 | Yes | 0.203 | No results found |
| IPI00333776.6 | Isoform 1 of Neuronal cell adhesion molecule | C | Q92823 | NRCAM | 4897 | 7q31 | Yes | 0.332 | No results found |
| IPI00027223.2 | Isocitrate dehydrogenase [NADP] cytoplasmic | both | O75874 | IDH1 | 3417 | 2q33.3 | No | 0.547 | ExoCarta_3417 |
| IPI00296180.3 | Urokinase-type plasminogen activator | both | P00749 | PLAU | 5328 | 10q24 | Yes | 0.650 | ExoCarta_5328 |
| IPI00304692.1 | Heterogeneous nuclear ribonucleoprotein G | both | P38159 | RBMX | 27316 | Xq26.3 | No | 0.276 | No results found |
| IPI00008274.7 | Isoform 1 of Adenylyl cyclase-associated protein 1 | C | Q01518 | CAP1 | 10487 | 1p34.2 | No | 0.429 | ExoCarta_10487 |
| IPI00298281.4 | Laminin subunit gamma-1 | both | P11047 | LAMC1 | 3915 | 1q31 | Yes | 0.284 | ExoCarta_3915 |
| IPI00019568.1 | Prothrombin (Fragment) | both | P00734 | F2 | 2147 | 11p11 | Yes | 0.618 | No results found |
| IPI00010277.1 | Isoform 1 of Tumor necrosis factor receptor superfamily member 12A | both | Q9NP84 | TNFRSF12A | 51330 | 16p13.3 | Yes | 0.716 | ExoCarta_51330 |
| IPI00008524.1 | Isoform 1 of Polyadenylate-binding protein 1 | both | P11940 | PABPC1 | 26986 | 8q22.2-q23 | No | 0.283 | No results found |
| IPI00026941.1 | Serine protease 23 | both | O95084 | PRSS23 | 11098 | 11q14.1 | Yes | 0.819 | ExoCarta_11098 |
| IPI00017704.3 | Coactosin-like protein | both | Q14019 | COTL1 | 23406 | 16q24.1 | No | 0.765 | ExoCarta_23406 |
| IPI00909764.1 | cDNA FLJ56650, highly similar to Matrilysin | both | B4DDW4 | - | - | - | Yes | 0.806 | No results found |
| IPI00216592.2 | Isoform C1 of Heterogeneous nuclear ribonucleoproteins C1/C2 | both | P07910 | HNRNPC | 3183 | 14q11.2 | No | 0.304 | ExoCarta_3183 |
| IPI00009030.1 | Isoform LAMP-2A of Lysosome-associated membrane glycoprotein 2 | both | P13473 | LAMP2 | 3920 | Xq24 | No | 0.680 | ExoCarta_3920 |
| IPI00032258.4 | Complement C4-A | both | P0C0L4 | C4A | 720 | 6p21.3 | Yes | 0.399 | ExoCarta_720 |
| IPI00000870.1 | Choriogonadotropin subunit beta | C | P01233 | CGB | 1082 | 19q13.32 | Yes | 0.408 | No results found |
| IPI00001611.1 | Isoform 1 of Insulin-like growth factor II | both | P01344 | IGF2 | 3481 | 11p15.5 | Yes | 0.864 | No results found |
| IPI00026087.1 | Barrier-to-autointegration factor | both | O75531 | BANF1 | 8815 | 11q13.1 | No | 0.551 | No results found |
| IPI00178440.3 | Elongation factor 1-beta | both | P24534 | EEF1B2 | 1933 | 2q33.3 | No | 0.562 | No results found |
| IPI00002745.1 | Cathepsin Z | both | Q9UBR2 | CTSZ | 1522 | 20q13.32 | Yes | 0.861 | No results found |
| IPI00032179.3 | Antithrombin-III | both | P01008 | SERPINC1 | 462 | 1q25.1 | Yes | 0.645 | No results found |
| IPI00022830.3 | Isoform 2 of NSFL1 cofactor p47 | both | Q9UNZ2 | NSFL1C | 55968 | 20p13 | No | 0.359 | No results found |
| IPI00005292.1 | Testican-1 | both | Q08629 | SPOCK1 | 6695 | 5q31.2 | Yes | 0.271 | ExoCarta_6695 |
| IPI00027628.1 | Interleukin-33 | both | O95760 | IL33 | 90865 | 9p24.1 | No | 0.323 | No results found |
| IPI00075248.11 | Calmodulin | both | P62158 | CALM1 | 801 | 14q32.11 | No | 0.676 | ExoCarta_801 |
| IPI00293276.10 | Macrophage migration inhibitory factor | both | P14174 | MIF | 4282 | 22q11.23 | No | 0.776 | ExoCarta_4282 |
| IPI00009276.2 | Endothelial protein C receptor precursor | both | Q9UNN8 | PROCR | 10544 | 20q11.2 | Yes | 0.916 | No results found |
| IPI00011865.2 | Isoform 2 of Platelet-derived growth factor D | both | Q9GZP0 | PDGFD | 80310 | 11q22.3 | Yes | 0.774 | No results found |
| IPI00385321.6 | Isoform 2 of Protein kinase C-binding protein 1 | C | Q9ULU4 | ZMYND8 | 23613 | 20q13.12 | No | 0.096 | No results found |
| IPI00014572.1 | SPARC | C | P09486 | SPARC | 6678 | 5q31.3-q32 | Yes | 0.942 | ExoCarta_6678 |
| IPI00026272.2 | Histone H2A type 1-B/E | C | P04908 | HIST1H2AB | 3012 | 6p22.1 | No | 0.536 | ExoCarta_8335 |
| IPI00295741.4 | Cathepsin B | both | P07858 | CTSB | 1508 | 8p22 | Yes | 0.770 | ExoCarta_1508 |
| IPI00334282.2 | Protein FAM3C | both | Q92520 | FAM3C | 10447 | 7q31 | No | 0.821 | No results found |
| IPI00848226.4 | Guanine nucleotide-binding protein subunit beta-2-like 1 | both | P63244 | GNB2L1 | 10399 | 5q35.3 | No | 0.465 | ExoCarta_10399 |
| IPI00030128.1 | Beta-1,4-galactosyltransferase 4 | C | O60513 | B4GALT4 | 8702 | 3q13.3 | Yes | 0.571 | No results found |
| IPI00294839.6 | ENTPD4 Lysyl oxidase homolog 2 | C | Q9Y4K0 | LOXL2 | 4017 | 8p21.3 | Yes | 0.711 | ExoCarta_4017 |
| IPI00012887.1 | Cathepsin L1 | both | P07711 | CTSL1 | 1514 | 9q21.33 | Yes | 0.514 | No results found |
| IPI00018140.3 | Isoform 1 of Heterogeneous nuclear ribonucleoprotein Q | M | O60506 | SYNCRIP | 10492 | 6q14-q15 | No | 0.081 | No results found |
| IPI00293867.7 | D-dopachrome decarboxylase | C | P30046 | DDT | 1652 | 22q11.23 | No | 0.479 | ExoCarta_1652 |
| IPI00024034.2 | Cadherin-4 | C | P55283 | CDH4 | 1002 | 20q13.3 | Yes | 0.258 | No results found |
| IPI00387168.2 | Proprotein convertase subtilisin/kexin type 9 | both | Q8NBP7 | PCSK9 | 255738 | 1p32.3 | Yes | 0.761 | ExoCarta_255738 |
| IPI00006952.3 | Beta-lactamase-like protein 2 | M | Q53H82 | LACTB2 | 51110 | 8q13.3 | No | 0.329 | No results found |
| IPI00298237.8 | cDNA FLJ56402, highly similar to Tripeptidyl-peptidase 1 | both | O14773 | TPP1 | 1200 | 11p15 | Yes | 0.746 | ExoCarta_1200 |
| IPI00016353.1 | Dickkopf-related protein 1 | both | O94907 | DKK1 | 22943 | 10q11.2 | Yes | 0.952 | ExoCarta_22943 |
| IPI00005614.6 | Isoform Long of Spectrin beta chain, brain 1 | both | Q01082 | SPTBN1 | 6711 | 2p21 | No | 0.177 | ExoCarta_6711 |
| IPI00011937.1 | Peroxiredoxin-4 | M | Q13162 | PRDX4 | 10549 | Xp22.11 | Yes | 0.724 | ExoCarta_10549 |
| IPI00031461.2 | cDNA FLJ60299, highly similar to Rab GDP dissociation inhibitor beta | C | P50395 | GDI2 | 2665 | 10p15 | No | 0.315 | ExoCarta_2665 |
| IPI00013917.3 | 40S ribosomal protein S12 | both | P25398 | RPS12 | 6206 | 6q23.2 | No | 0.661 | No results found |
| IPI00012066.2 | poly(rC) binding protein 2 isoform b | both | Q15366 | PCBP2 | 5094 | 12q13.12-q13.13 | No | 0.543 | No results found |
| IPI00025869.1 | Alpha-galactosidase A | both | P06280 | GLA | 2717 | Xq22 | Yes | 0.713 | No results found |
| IPI00296913.1 | ADP-sugar pyrophosphatase | M | Q9UKK9 | NUDT5 | 11164 | 10p14 | No | 0.188 | ExoCarta_11164 |
| IPI00007257.4 | Isoform 2 of Calsyntenin-1 (Fragment) | M | O94985 | CLSTN1 | 22883 | 1p36.22 | Yes | 0.436 | ExoCarta_22883 |
| IPI00005705.1 | Isoform Gamma-1 of Serine/threonine-protein phosphatase PP1-gamma catalytic subunit | both | P36873 | PPP1CC | 5501 | 12q24.1-q24.2 | No | 0.480 | ExoCarta_5501 |
| IPI00015842.1 | Reticulocalbin-1 | both | Q15293 | RCN1 | 5954 | 11p13 | Yes | 0.851 | No results found |
| IPI00025418.2 | Isoform 1 of Collagen alpha-1(VII) chain | C | Q02388 | COL7A1 | 1294 | 3p21.1 | Yes | 0.065 | ExoCarta_1294 |
| IPI00220362.5 | 10 kDa heat shock protein, mitochondrial | both | P61604 | HSPE1 | 3336 | 2q33.1 | No | 0.570 | ExoCarta_3336 |
| IPI00411680.9 | protein-L-isoaspartate (D-aspartate) O-methyltransferase 1 | both | P22061 | PCMT1 | 5110 | 6q24-q25 | No | 0.515 | No results found |
