# Supplementary material for: Global secretome characterization of A549 human alveolar epithelial carcinoma cells during Mycoplasma pneumoniae infection
Source: BMC Microbiol. 2014 Feb 7;14:27. doi: 10.1186/1471-2180-14-27 (PMC3922035; doi:10.1186/1471-2180-14-27)
Supplement: Additional file 6: Table S2 — Differentially expressed proteins identified in the secretome of Mycoplasma pneumoniae-infected A549 and untreated A549 cells. [file 1471-2180-14-27-S6.doc]

| **Table S2. Differentially expressed proteins identified in the media of *Mycoplasma pneumoniae*-infected A549 and untreated A549** | | | | | |
| --- | --- | --- | --- | --- | --- |
| Protein name | IPI accession number | Number of peptides used for quatitation | Peptide sequence | Ratio: Mp-infected A549/untreated A549 | Means ratio±SD (Coefficient of variation) |
| **Upregulated** |  |  |  |  |  |
| Retinal dehydrogenase 1 | IPI00218914.5 | 13 | K.KYILGNPLTPGVTQGPQIDK.E | 2.65 | 1.95±0.43(21.93%) |
|  |  |  | K.IFINNEWHDSVSGK.K | 2.02 |  |
|  |  |  | K.LYSNAYLNDLAGCIK.T | 2.99 |  |
|  |  |  | K.ILDLIESGK.K | 1.98 |  |
|  |  |  | K.RANNTFYGLSAGVFTK.D | 1.96 |  |
|  |  |  | K.ILDLIESGK.K | 1.95 |  |
|  |  |  | K.SLDDVIK.R | 1.88 |  |
|  |  |  | K.YILGNPLTPGVTQGPQIDK.E | 1.77 |  |
|  |  |  | R.TIPIDGNFFTYTR.H | 1.75 |  |
|  |  |  | K.IFINNEWHDSVSGK.K | 1.71 |  |
|  |  |  | R.QAFQIGSPWR.T | 1.61 |  |
|  |  |  | K.PAEQTPLTALHVASLIK.E | 1.53 |  |
|  |  |  | K.LECGGGPWGNK.G | 1.52 |  |
| Aldo-ketoreductase family 1 member B10 | IPI00105407.1 | 9 | K.TAAQVLIR.F | 3.88 | 2.13±0.77(36.10%) |
|  |  |  | K.LSDEEMATILSFNR.N | 2.86 |  |
|  |  |  | K.LLNKPGLK.Y | 2.09 |  |
|  |  |  | K.REDLFIVSK.L | 1.87 |  |
|  |  |  | K.LSYLDVYLIHWPQGFK.S | 1.84 |  |
|  |  |  | K.ALGVSNFSHFQIEK.L | 1.84 |  |
|  |  |  | R.IVENIQVFDFK.L | 1.68 |  |
|  |  |  | K.SGDDLFPK.D | 1.55 |  |
|  |  |  | K.VAIDAGYR.H | 1.54 |  |
| Tubulin alpha-1B chain | IPI00930688.1 | 6 | K.TIGGGDDSFNTFFSETGAGK.H | 1.73 | 1.60±0.08(5.23%) |
|  |  |  | R.AVFVDLEPTVIDEVR.T | 1.59 |  |
|  |  |  | K.EIIDLVLDR.I | 1.57 |  |
|  |  |  | R.SIQFVDWCPTGFK.V | 1.52 |  |
|  |  |  | R.LISQIVSSITASLR.F | 1.51 |  |
|  |  |  | R.IHFPLATYAPVISAEK.A | 1.65 |  |
| Isoform 1 of Triosephosphateisomerase | IPI00797270.4 | 6 | K.VVLAYEPVWAIGTGK.T | 1.68 | 1.66±0.13(7.66%) |
|  |  |  | K.QSLGELIGTLNAAK.V | 1.67 |  |
|  |  |  | K.VPADTEVVCAPPTAYIDFAR.Q | 1.64 |  |
|  |  |  | K.SNVSDAVAQSTR.I | 1.59 |  |
|  |  |  | R.IIYGGSVTGATCK.E | 1.51 |  |
|  |  |  | K.IAVAAQNCYK.V | 1.89 |  |
| Alpha-enolase | IPI00465248.5 | 5 | K.FGANAILGVSLAVCK.A | 2.93 | 1.94±0.56(28.58%) |
|  |  |  | K.DATNVGDEGGFAPNILENK.E | 1.77 |  |
|  |  |  | K.FTASAGIQVVGDDLTVTNPK.R | 1.76 |  |
|  |  |  | R.IEEELGSK.A | 1.65 |  |
|  |  |  | R.YISPDQLADLYK.S | 1.61 |  |
| Profilin-1 | IPI00216691.5 | 4 | R.SSFYVNGLTLGGQK.C | 2.30 | 1.82±0.34(18.98%) |
|  |  |  | K.STGGAPTFNVTVTK.T | 1.59 |  |
|  |  |  | K.TFVNITPAEVGVLVGK.D | 1.55 |  |
|  |  |  | K.TLVLLMGK.E | 1.82 |  |
| Clusterin | IPI00291262.3 | 3 | R.RPHFFFPK.S | 3.17 | 2.20±0.84(38.40%) |
|  |  |  | K.LFDSDPITVTVPVEVSRK.N | 1.74 |  |
|  |  |  | K.CREILSVDCSTNNPSQAK.L | 1.68 |  |
| Aldose reductase | IPI00413641.7 | 3 | K.REELFIVSK.L | 1.70 | 1.63±0.08(4.68%) |
|  |  |  | R.NLVVIPK.S | 1.65 |  |
|  |  |  | K.LDYLDLYLIHWPTGFKPGK.E | 1.55 |  |
| Plasminogen activator inhibitor 1 （PAI） | IPI00007118.1 | 2 | R.VFQQVAQASK.D | 4.15 | 3.34±1.15(34.56%) |
|  |  |  | R.LVLVNALYFNGQWK.T | 2.52 |  |
| YWHAZ 14-3-3 protein zeta/delta | IPI00021263.3 | 2 | K.DSTLIMQLLR.D | 2.90 | 2.33±0.81(34.60%) |
|  |  |  | K.SVTEQGAELSNEER.N | 1.76 |  |
| 14-3-3 protein epsilon | IPI00000816.1 | 2 | K.EAAENSLVAYK.A | 2.06 |  |
|  |  |  | K.LICCDILDVLDK.H | 1.64 | 1.85±0.30(16.05%) |
| Peptidyl-prolylcis-trans isomerase A | IPI00419585.9 | 2 | K.FEDENFILK.H | 2.05 | 1.78±0.38(21.45%) |
|  |  |  | M.VNPTVFFDIAVDGEPLGR.V | 1.51 |  |
| Ubiquitin-40S ribosomal protein S27a | IPI00179330.6 | 2 | K.ESTLHLVLR.L | 2.28 | 1.96±0.45(23.09%) |
|  |  |  | K.TITLEVEPSDTIENVK.A | 1.64 |  |
| Vimentin | IPI00418471.6 | 2 | R.EYQDLLNVK.M | 2.24 | 2.20±0.06(2.57%) |
|  |  |  | K.NLQEAEEWYK.S | 2.16 |  |
| Thioredoxin | IPI00216298.6 | 2 | K.EKLEATINELV.- | 1.86 | 1.75±0.16(8.89%) |
|  |  |  | K.TAFQEALDAAGDK.L | 1.64 |  |
| Insulin-like growth factor-binding protein 4 | IPI00305380.3 | 2 | K.GELDCHQLADSFR.E | 1.80 | 1.65±0.21(12.86%) |
|  |  |  | K.QCHPALDGQR.G | 1.50 |  |
| Isoform Sap-mu-0 of Proactivator polypeptide | IPI00012503.1 | 2 | K.QEILAALEK.G | 1.74 | 1.66±0.12(7.26%) |
|  |  |  | K.QEILAALEK.G | 1.57 |  |
| Keratin, type II cytoskeletal | IPI00554648.3 | 2 | K.YEELQSLAGK.H | 1.70 | 1.77±0.10(5.59%) |
|  |  |  | R.LEGLTDEINFLR.Q | 1.84 |  |
| Peroxiredoxin-1 | IPI00000874.1 | 2 | K.DISLSDYK.G | 1.67 | 1.61±0.09(5.73%) |
|  |  |  | R.LVQAFQFTDK.H | 1.54 |  |
| Cadherin-2 | IPI00290085.2 | 2 | FLIYAQDK.E | 1.62 | 1.62±0.00(0.00%) |
|  |  |  | K.DVHEGQPLLNVK.F | 1.62 |  |
| Isoform 1 of Filamin-B | IPI00289334.1 | 2 | R.EAGAGGLSIAVEGPSK.A | 1.59 | 2.20±0.86(39.21%) |
|  |  |  | R.LVSPGSANETSSILVESVTR.S | 2.81 |  |
| Beta-2-microglobulin | IPI00004656.3 | 2 | K.VEHSDLSFSK.D | 1.54 | 2.12±0.81(38.45%) |
|  |  |  | R.VNHVTLSQPK.I | 2.69 |  |
| L-lactate dehydrogenase B chain | IPI00219217.3 | 2 | K.DYSVTANSK.I | 2.51 | 2.63±0.16(6.20%) |
|  |  |  | K.IVVVTAGVR.Q | 2.74 |  |
| Isoform Long of 14-3-3 protein beta/alpha | IPI00216318.5 | 1 | R.VISSIEQK.T ! R.VLSSIEQK.S | 1.51 | 1.51 |
| Galectin-3-binding protein | IPI00023673.1 | 1 | R.IYTSPTWSAFVTDSSWSAR.K | 2.83 | 2.83 |
| HSPB1 Heat shock protein beta-1 | IPI00025512.2 | 1 | R.LFDQAFGLPR.L | 1.95 | 1.95 |
| Ubiquitin-like modifier-activating enzyme 1 | IPI00645078.1 | 1 | K.YFLVGAGAIGCELLK.N | 1.91 | 1.91 |
| Annexin A2 | IPI00455315.4 | 1 | K.GVDEVTIVNILTNR.S | 1.87 | 1.87 |
| Metalloproteinase inhibitor 2 | IPI00027166.1 | 1 | R.SDGSCAWYR.G | 1.87 | 1.87 |
| Transforming growth factor-beta-induced protein ig-h3 | IPI00018219.1 | 1 | K.YHIGDEILVSGGIGALVR.L | 1.86 | 1.86 |
| Aspartate aminotransferase | IPI00219029.3 | 1 | M.APPSVFAEVPQAQPVLVFK.L | 1.84 | 1.84 |
| Cystatin-C | IPI00032293.1 | 1 | K.TQPNLDNCPFHDQPHLK.R | 1.80 | 1.80 |
| Thioredoxin domain-containing protein 17 | IPI00646689.1 | 1 | R.YEEVSVSGFEEFHR.A | 1.78 | 1.78 |
| Glucose-6-phosphate isomerase | IPI00027497.5 | 1 | R.AVLHVALR.N | 1.77 | 1.77 |
| Isoform 5 of Thioredoxinreductase 1 | IPI00554786.5 | 1 | R.FLIATGERPR.Y | 1.74 | 1.74 |
| Isoform 1 of Disintegrin and metalloproteinase domain-containing protein 9 | IPI00440932.1 | 1 | K.DLLPEDFVVYTYNK.E | 1.72 | 1.72 |
| cDNA FLJ59142, highly similar to Epididymal secretory protein E1 | IPI00940960.  1 | 1 | K.DCGSVDGVIK.E | 1.72 | 1.72 |
| Calmodulin | IPI00075248.11 | 1 | K.EAFSLFDK.D | 1.71 | 1.71 |
| Isoform Long of Beta-1,4-galactosyltransferase 1 | IPI00215767.1 | 1 | R.QQLDYGIYVINQAGDTIFNR.A | 1.71 | 1.71 |
| Keratin, type II cytoskeletal 7 | IPI00306959.10 | 1 | K.VDALNDEINFLR.T | 1.69 | 1.69 |
| Elongation factor 1-beta | IPI00178440.3 | 1 | K.SPAGLQVLNDYLADK.S | 1.69 | 1.69 |
| Endothelial protein C receptor precursor | IPI00009276.2 | 1 | R.TQSGLQSYLLQFHGLVR.L | 1.67 | 1.67 |
| Isoform A1-B of Heterogeneous nuclear ribonucleoprotein A1 | IPI00215965.2 | 1 | K.LFIGGLSFETTDESLR.S | 1.65 | 1.65 |
| Cathepsin D | IPI00011229.1 | 1 | K.LLDIACWIHHK.Y | 1.62 | 1.62 |
| Isoform 2 of Platelet-derived growth factor D | IPI00011865.2 | 1 | K.IAEFDTVEDLLK.Y | 1.62 | 1.62 |
| Moesin | IPI00219365.3 | 1 | R.NISFNDK.K | 1.62 | 1.62 |
| Cofilin-1 | IPI00012011.6 | 1 | K.NIILEEGK.E | 1.62 | 1.62 |
| Isoform 2 of Calumenin | IPI00045396.1 | 1 | K.YDLFVGSQATDFGEALVR.H | 1.61 | 1.61 |
| Isoform 1 of Fibronectin | IPI00022418.1 | 1 | R.DLQFVEVTDVK.V | 1.57 | 1.57 |
| L-lactate dehydrogenase | IPI00217966.8 | 1 | K.DYNVTANSK.L | 1.54 | 1.54 |
| Cathepsin L1 | IPI00012887.1 | 1 | K.VFQEPLFYEAPR.S | 1.54 | 1.54 |
| Keratin, type I cytoskeletal 18 | IPI00554788.5 | 1 | K.ASLENSLR.E | 1.54 | 1.54 |
| Galectin-1 | IPI00219219.3 | 1 | K.DSNNLCLHFNPR.F | 1.52 | 1.52 |
| LOC284889 Macrophage migration inhibitory factor | IPI00293276.10 | 1 | K.LLCGLLAER.L | 1.94 | 1.94 |
| Interleukin-33 | IPI00027628.1 | 1 | K.TGRKHKRH.L | 1.69 | 1.69 |
| ADP-sugar pyrophosphatase | IPI00296913.1 | 1 | EQTADGVAVIPVLQR.T | ＞1.50^a^ | ＞1.50^a^ |
| Isoform 2 of Calsyntenin-1 (Fragment) | IPI00007257.4 | 1 | K.DYSFTIQAYDCGK.G | ＞1.50^a^ | ＞1.50^a^ |
| Isoform APP770 of Amyloid beta A4 protein (Fragment | IPI00006608.1 | 1 | R.EVCSEQAETGPCR.A | ＞1.50^a^ | ＞1.50^a^ |
| Protein disulfide-isomerase | IPI00010796.1 | 2 | K.ILFIFIDSDHTDNQR.I | ＞1.50^a^ | ＞1.50^a^ |
| Purine nucleoside phosphorylase | IPI00017672.4 | 1 | R.VFGFSLITNK.V | ＞1.50^a^ | ＞1.50^a^ |
| Isoform 1 of Tropomyosin alpha-4 chain | IPI00010779.4 | 1 | R.LATALQK.L | ＞1.50^a^ | ＞1.50^a^ |
| 6-phosphogluconate dehydrogenase | IPI00219525.10 | 1 | K.WTAISALEYGVPVTLIGEAVFAR.C | ＞1.50^a^ | ＞1.50^a^ |
| Peroxiredoxin-4 | IPI00011937.1 | 1 | K.DYGVYLEDSGHTLR.G | ＞1.50^a^ | ＞1.50^a^ |
| Isoform 1 of Heterogeneous nuclear ribonucleoprotein Q | IPI00018140.3 | 1 | K.EFNEDGALAVLQQFK.D | ＞1.50^a^ | ＞1.50^a^ |
| Beta-lactamase-like protein 2 | IPI00006952.3 | 1 | R.EQQILTLFR.E | ＞1.50^a^ | ＞1.50^a^ |
| **downregulated** |  |  |  |  |  |
| Follistatin-related protein 1 | IPI00029723.1 | 5 | K.NFDNGDSRLDSSEFLK.F | 0.46 | 0.57±0.07(11.50%) |
|  |  |  | K.GEPTCLCIEQCK.P | 0.57 |  |
|  |  |  | K.TYLNHCELHR.D | 0.58 |  |
|  |  |  | K.LSFQEFLK.C | 0.62 |  |
|  |  |  | K.ICANVFCGAGR.E | 0.62 |  |
| Glutathione S-transferase P | IPI00219757.13 | 4 | K.AFLASPEYVNLPINGNGK.Q | 0.46 | 0.54±0.08(14.62%) |
|  |  |  | K.ALPGQLKPFETLLSQNQGGK.T | 0.49 |  |
|  |  |  | K.FQDGDLTLYQSNTILR.H | 0.60 |  |
|  |  |  | K.ASCLYGQLPK.F | 0.62 |  |
| Nucleoside diphosphate kinase B | IPI00795292.1 | 4 | R.GDFCIQVGR.N | 0.62 | 0.65±0.02(3.23%) |
|  |  |  | K.DRPFFAGLVK.Y | 0.64 |  |
|  |  |  | R.GLVGEIIK.R | 0.65 |  |
|  |  |  | R.TFIAIKPDGVQR.G | 0.67 |  |
| Alpha-actinin-4 | IPI00013808.1 | 3 | K.LSGSNPYTTVTPQIINSK.W | 0.66 | 0.59±0.07(11.11%) |
|  |  |  | R.VGWEQLLTTIAR.T | 0.58 |  |
|  |  |  | K.GYEEWLLNEIR.R | 0.53 |  |
| 14-3-3 protein gamma | IPI00220642.7 | 2 | K.NVTELNEPLSNEER.N | 0.48 |  |
|  |  |  | R.YLAEVATGEK.R | 0.31 | 0.40±0.12(30.43%) |
| Transketolase | IPI00643920.3 | 2 | K.AVELAANTK.G | 0.59 | 0.62±0.04(6.84%) |
|  |  |  | K.LDNLVAILDINR.L | 0.65 |  |
| Isoform Non-muscle of Myosin light polypeptide 6 | IPI00335168.9 | 2 | K.EAFQLFDR.T | 0.62 | 0.56±0.09(16.56%) |
|  |  |  | K.DQGTYEDYVEGLR.V | 0.49 |  |
| Basement membrane-specific heparan sulfate proteoglycan core protein | IPI00024284.5 | 2 | K.DFISLGLQDGHLVFR.Y | 0.63 | 0.63±0.00(0.00%) |
|  |  |  | R.VVPYFTQTPYSFLPLPTIK.D | 0.63 |  |
| Isoform 2 of Filamin-A | IPI00302592.2 | 2 | R.AEAGVPAEFSIWTR.E | 0.23 | 0.20±0.05(25.38%) |
|  |  |  | R.VTYCPTEPGNYIINIK.F | 0.16 |  |
| Antithrombin-III | IPI00032179.3 | 1 | K.TSDQIHFFFAK.L | 0.22 | 0.22 |
| Testican-1 | IPI00005292.1 | 1 | K.SLLGAFIPR.C | 0.49 | 0.49 |
| 14-3-3 protein theta | IPI00018146.1 | 1 | K.AVTEQGAELSNEER.N | 0.31 | 0.31 |
| plasminogen activator inhibitor type 1, member 2 isoform b precursor | IPI00914848.1 | 1 | R.LVLVNAVYFK.G | 0.34 | 0.34 |
| Isoform Long of Spectrin beta chain, brain 1 | IPI00005614.6 | 1 | R.LTTLELLEVR.R | 0.55 | 0.55 |
| Malate dehydrogenase | IPI00291006.2 | 1 | K.IFGVTTLDIVR.A | 0.56 | 0.56 |
| Isoform 1 of Probable G-protein coupled receptor 126 | IPI00217481.3 | 1 | R.ISVVIQNILR.H | 0.59 | 1.71 |
| Metalloproteinase inhibitor 1 | IPI00032292.1 | 1 | K.GFQALGDAADIR.F | 0.59 | 0.59 |
| Fatty acid-binding protein, epidermal | IPI00007797.3 | 1 | K.ELGVGIALRK.M | 0.59 | 0.59 |
| Isoform 1 of Polypyrimidine tract-binding protein 1 | IPI00179964.5 | 1 | R.GQPIYIQFSNHK.E | 0.60 | 0.60 |
| Isoform M2 of Pyruvate kinase isozymes M1/M2 | IPI00479186.7 | 1 | K.GADFLVTEVENGGSLGSK.K | 0.60 | 0.60 |
| Adenosylhomocysteinase | IPI00012007.6 | 1 | R.GISEETTTGVHNLYK.M | 0.61 | 0.61 |
| Coactosin-like protein | IPI00017704.3 | 1 | K.FALITWIGENVSGLQR.A | 0.61 | 0.61 |
| Protein AMBP | IPI00022426.1 | 1 | R.AFIQLWAFDAVK.G | 0.61 | 0.61 |
| Protein S100-A11 | IPI00013895.1 | 1 | R.CIESLIAVFQK.Y | 0.61 | 0.61 |
| Ubiquitin carboxyl-terminal hydrolase isozyme L1 | IPI00018352.1 | 1 | R.LGVAGQWR.F | 0.62 | 0.62 |
| HSPA5 protein | IPI00003362.2 | 1 | K.NQLTSNPENTVFDAK.R | 0.63 | 0.63 |
| Destrin | IPI00473014.5 | 1 | R.YALYDASFETK.E | 0.64 | 0.64 |
| Annexin A1 | IPI00218918.5 | 1 | K.GVDEATIIDILTK.R | 0.66 | 0.66 |
| Aldo-ketoreductase family 1 member C3 | IPI00291483.3 | 1 | K.RTPALIALR.Y | 0.66 | 0.66 |
| Flavinreductase | IPI00783862.2 | 1 | K.TVAGQDAVIVLLGTR.N | 0.66 | 0.66 |
| Malate dehydrogenase, cytoplasmic | IPI00291005.8 | 1 | R.VLVTGAAGQIAYSLLYSIGNGSVFGK.D | 0.66 | 0.66 |
| Isoform 1 of Proteasome subunit alpha type-7 | IPI00024175.3 | 1 | K.ALLEVVQSGGK.N | 0.66 | 0.66 |
| Cytochrome c oxidase subunit 6B1 | IPI:IPI00797738.1 | 1 | R.VYQSLCPTSWVTDWDEQR.A | 0.64 | 0.64 |
| Proproteinconvertasesubtilisin/kexin type 9 | IPI00387168.2 | 1 | K.SQLVQPVGPLVVLLPLAGGYSR.V | 0.59 | 0.59 |
| Isoform 3 of Heat shock protein 105 kDa | IPI00513743.1 | 1 | R.FVVQNVSAQK.D | 0.51 | 0.51 |
| Serine/threonine-protein phosphatase PP1-gamma catalytic subunit | IPI00005705.1 | 1 | K.QSLETICLLLAYK.I | 0.50 | 0.50 |
| Isoform Long of Glucose-6-phosphate 1-dehydrogenase | IPI00216008.4 | 1 | R.DNIACVILTFK.E | 0.42 | 0.42 |
| Reticulocalbin-1 | IPI00015842.1 | 1 | R.HLVYESDKNK.D ! R.HLVYESDQNK.D | 0.42 | 0.42 |
| Isoform Mitochondrial of Glutathione reductase, mitochondrial | IPI00016862.1 | 1 | K.ALLTPVAIAAGRK.L | 0.15 | 0.15 |
| Isoform 1 of Neuronal cell adhesion molecule | IPI00333776.6 | 1 | R.ISWLTNGVPIEIAPDDPSR.K | ＜0.67^b^ | ＜0.67^b^ |
| Protein dpy-30 homolog | IPI00028109.1 | 1 | R.AYLDQTVVPILLQGLAVLAK.E | ＜0.67^b^ | ＜0.67^b^ |
| D-dopachrome decarboxylase | IPI00293867.7 | 1 | R.FFPLESWQIGK.I | ＜0.67^b^ | ＜0.67^b^ |
| SPARC | IPI00014572.1 | 1 | R.FFETCDLDNDK.Y | ＜0.67^b^ | ＜0.67^b^ |
| Histone H2A type 1-B/E | IPI00026272.2 | 1 | R.VTIAQGGVLPNIQAVLLPK.K | ＜0.67^b^ | ＜0.67^b^ |
| Rab GDP dissociation inhibitor beta | IPI00031461.2 | 1 | K.DLGTESQIFISR.T | ＜0.67^b^ | ＜0.67^b^ |
| Cadherin-4 | IPI00024034.2 | 1 | K.VGADGTVFATR.E | ＜0.67^b^ | ＜0.67^b^ |
| Isoform 1 of Far upstream element-binding protein 1 | IPI00375441.2 | 1 | R.IAQITGPPDR.C | ＜0.67^b^ | ＜0.67^b^ |
| Isoform 1 of Collagen alpha-1(VII) chain | IPI00025418.2 | 1 | R.AVTGSTEACHPFVYGGCGGNANR.F | ＜0.67^b^ | ＜0.67^b^ |
| ^a^ only found in control cells | | | | | |
| ^b^ only found in Mp-infected A549 cells | | | | | |
