# Supplementary material for: Global secretome characterization of A549 human alveolar epithelial carcinoma cells during Mycoplasma pneumoniae infection
Source: BMC Microbiol. 2014 Feb 7;14:27. doi: 10.1186/1471-2180-14-27 (PMC3922035; doi:10.1186/1471-2180-14-27)
Supplement: Additional file 8: Table S3 — Primers used for PCR amplification. [file 1471-2180-14-27-S8.doc]

| **Table S3.** Primers used for PCR amplification. | | |
| --- | --- | --- |
| Primer designation | | Sequence (5'→3') |
| SERPINC1 | Forward | AGCTCACTGTTCTGGTGCTGGTTA |
|  | Reverse | TTGTGTTCTCAGGGCTGAACTTTG |
| IL-33 | Forward | GTTGCATGCCAACAACAAGGA |
|  | Reverse | GCATTCAAATGAAACACAGTTGGAG |
| MIF | Forward | ACAGTGGTGTCCGAGAAGTCAG |
|  | Reverse | TGGCAGAAGGACCAGGAGA |
| LGALS3BP | Forward | CCTGAACCTCACCGAGGATACCTA |
|  | Reverse | CACTCCAGGAACTGTCTGTCACAA |
| CTSL1 | Forward | TTGGCTACGGATTTGAAAGCAC |
|  | Reverse | CCGGTCTTTGGCCATCTTTAC |
| CST3 | Forward | CCCAGCCCAACTTGGACAAC |
|  | Reverse | CTAGGCGTCCTGACAGGTGGAT |
| GPI | Forward | CATCAACTGCTTTGGGTGTGAGA |
|  | Reverse | CGGGTTCCAGATTTGGTGATG |
| B4GALT1 | Forward | CGGCAGGAGCACCTCAAGTA |
|  | Reverse | TGAGGTCCACGTCACTAAACACAA |
| AMBP | Forward | TGCATGGGCAACGGTAACA |
|  | Reverse | TGACAGCATCAAATGCCCAGA |
| TIMP2 | Forward | GACGGCAAGATGCACATCAC |
|  | Reverse | GAGATGTAGCACGGGATCATGG |
| TXN | Forward | CAGCCAAGATGGTGAAGCAGA |
|  | Reverse | TTTATCACCTGCAGCGTCCAA |
| ADAM9 | Forward | ATTCCAAAGCCTGATGAAGCCTA |
|  | Reverse | TTCGCAGCAAGGGTCCAA |
| SPOCK1 | Forward | CCCAGAAACACTGGGTTGGAC |
|  | Reverse | TTGCCAGTAGAACAAGCATGGAA |
| IGFBP4 | Forward | CCCACTCCCAAAGCTCAGACT |
|  | Reverse | TGCAACAACCAGACCTAAAGGA |
| SERPINE1 | Forward | GATGGCTCAGACCAACAAGTTCA |
|  | Reverse | GGGCAGTTCCAGGATGTCGTA |
| LGALS1 | Forward | GAGGTGGCTCCTGACGCTAA |
|  | Reverse | CCTTGCTGTTGCACACGATG |
